# Innate immune cells in the pathophysiology of calcific aortic valve disease: lessons to be learned from atherosclerotic cardiovascular disease?

**DOI:** 10.1007/s00395-022-00935-6

**Published:** 2022-05-17

**Authors:** Wieteke Broeders, Siroon Bekkering, Saloua El Messaoudi, Leo A. B. Joosten, Niels van Royen, Niels P. Riksen

**Affiliations:** 1 Department of Internal Medicine and Radboud Institute for Molecular Life Sciences (RIMLS), Radboud University Medical Centre, Geert Grooteplein Zuid 8, 6525 GA Nijmegen, The Netherlands; 2 Department of Cardiology, Radboud University Medical Centre, Nijmegen, The Netherlands; 3 Department of Medical Genetics, Iuliu Hațieganu University of Medicine and Pharmacy, Cluj-Napoca-Napoca, Romania

**Keywords:** Calcific aortic valve disease, Atherosclerotic cardiovascular disease, Inflammation, Innate immune cells, Trained immunity, Clonal haematopoiesis of indeterminate potential

## Abstract

Calcific aortic valve disease (CAVD) is the most common valvular disease in the developed world with currently no effective pharmacological treatment available. CAVD results from a complex, multifactorial process, in which valvular inflammation and fibro-calcific remodelling lead to valve thickening and cardiac outflow obstruction. The exact underlying pathophysiology of CAVD is still not fully understood, yet the development of CAVD shows many similarities with the pathophysiology of atherosclerotic cardiovascular disease (ASCVD), such as coronary artery disease. Innate immune cells play a crucial role in ASCVD and might also play a pivotal role in the development of CAVD. This review summarizes the current knowledge on the role of innate immune cells, both in the circulation and in the aortic valve, in the development of CAVD and the similarities and differences with ASCVD. Trained immunity and clonal haematopoiesis of indeterminate potential are proposed as novel immunological mechanisms that possibly contribute to the pathophysiology of CAVD and new possible treatment targets are discussed.

## Introduction

Calcific aortic valve disease (CAVD) is the most common type of valvular heart disease in the Western world and is characterized by valvular inflammation, fibrosis and calcification. It is the leading cause of aortic valve stenosis and, ultimately, it can cause angina, syncope, heart failure and sudden cardiac death [27]. One in four people over 65 years suffer from aortic valve sclerosis of which 10–15% progresses to aortic valve stenosis [114]. Once symptomatic, untreated patients have a poor prognosis with a 2- and 5-year survival rate of 50% and 25%, respectively [114]. CAVD has a major impact on health care and this is expected to increase in the coming decades due to the ageing population [16]. Currently, no effective pharmacological treatment is available to prevent CAVD or slow down disease progression.

Traditionally, the development of CAVD was seen as a passive, degenerative process, but nowadays it is increasingly recognized as an active, multifactorial process with an important role for activation of the innate immune system. Importantly, this process appears to have many similarities with the pathophysiology of atherosclerosis and the pathophysiological process underlying atherosclerotic cardiovascular disease (ASCVD), such as coronary artery disease (CAD) [19, 82]. However, the exact underlying pathophysiology of CAVD remains incompletely understood, which hampers target-specific development of pharmacotherapy.

In this review, we discuss the role of innate immune cells, and in particular the role of monocytes, in the development of CAVD, and its similarities and differences with ASCVD. After a brief comparison of the overall pathophysiology of CAVD and ASCVD, we discuss in detail the current knowledge on valvular and systemic inflammation and innate immune cells in the development and progression of CAVD and the pivotal role of oxidized lipids. For each component, we systematically compare its role in CAVD and ASCVD. Furthermore, we propose two novel immunological mechanisms that might contribute to innate immune system activation in CAVD, namely trained immunity and clonal haematopoiesis of indeterminate potential (CHIP). Finally, we discuss how this knowledge might deliver novel therapeutic targets for the treatment of CAVD.

## Summary of the pathophysiology of CAVD

The aortic valve is tricuspid, although 1–2% of individuals have a bicuspid or even a unicuspid or quadricuspid valve. Aortic valve leaflets consist of valvular endothelial cells (VECs), valvular interstitials cells (VICs) and valvular extracellular matrix (VECM) [119]. VECs cover the leaflets and regulate valve permeability and homeostasis. The valvular interstitium is composed of three layers: the laminae fibrosa (aortic side), spongiosa and ventricularis. VICs are found throughout the interstitium and regulate valve remodelling via the synthesis and degradation of VECM components. VICs are quiescent and have characteristics similar to fibroblasts in the homeostatic state [115]. Furthermore, healthy valves contain few resident macrophages, mast cells and dendritic cells as well as a small number of myofibroblast-like cells [40, 113].

**Fig. 1:**
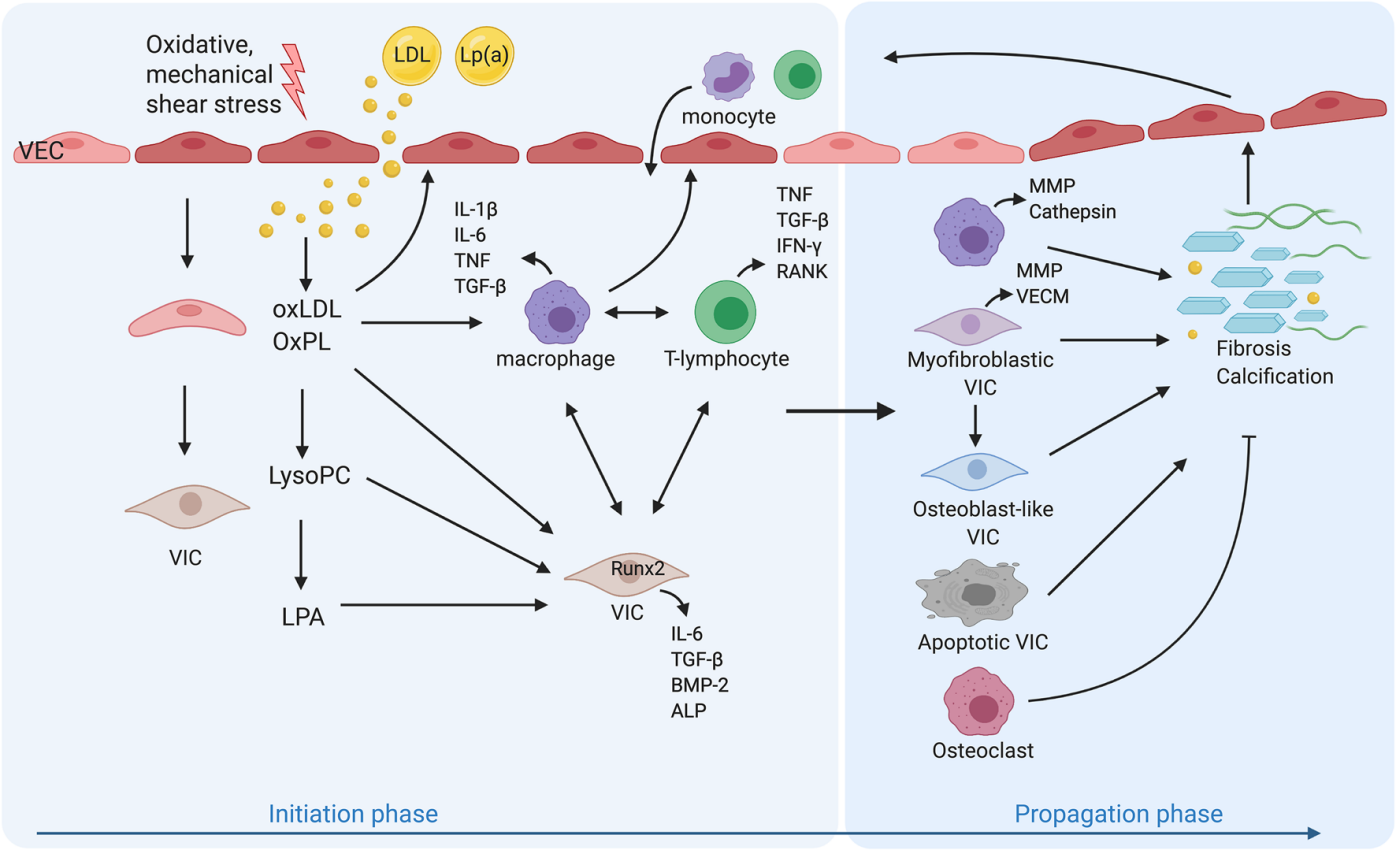
The pathogenesis of calcific aortic valve disease. In the initiation phase, valvular endothelial cells (VECs) are activated by oxidative, mechanical or shear stress, leading to increased valvular permeability. This results in infiltration of circulating lipids (lipoprotein (a) (Lp(a)) and low-density lipoprotein (LDL)) and immune cells, such as monocytes, neutrophils and lymphocytes. The oxidized LDL (oxLDL) and Lp(a) contain oxidized phospholipids (OxPL), which both activate macrophages and T lymphocytes and stimulate the release of various pro-inflammatory molecules that activate other immune cells, VECs, and valvular interstitial cells (VICs). The oxidized lipids also directly activate VEC and increase the expression of adhesion molecules, prompting the recruitment of more immune cells. OxPL are transformed into lysophosphatidylcholine (LysoPC) by lipoprotein-phospholipase A_2_ (Lp-PLA_2_), which is subsequently converted into lysophosphatidic acid (LPA) by autotaxin (ATX). LPA then activates VICs, triggering an NF-κB-regulated inflammatory cascade, which results in increased expression of bone morphogenic protein (BMP) 2, IL-6 and Runt-related transcription factor 2 (Runx2) and secretion of alkaline phosphatase (ALP). Additionally, the OxPL derivate LysoPC induces apoptosis in VICs. In the propagation phase, VICs differentiate into a myofibroblastic or osteoblast-like phenotype upon stimulation by the pro-inflammatory molecules and promote fibrosis and calcification, respectively. The activated macrophages and myofibroblastic VICs secrete matrix remodelling proteins and valvular extracellular matrix (VECM) components. The continuous redeposition and destruction of VECM creates valvular stiffness. The chronic inflammation stimulates apoptosis of macrophages and VICs and the release of extracellular vesicles, including apoptotic bodies, which both promote the continuous deposition of microcalcifications and crystals. Osteoblast-like VICs induce biomineralization in a way akin to osteogenesis. T lymphocytes stimulate proinflammatory polarization of macrophages and the osteogenic differentiation of VICs. IFN-γ, produced by T lymphocytes, inhibits the function of macrophage-derived osteoclasts. Together, these processes create accumulation of calcium and leaflet stiffening, creating more mechanical stress and thereby prompting more calcium deposition, establishing a self-perpetuating cycle which eventually leads to valvular outflow obstruction

The current proposed pathophysiological process of CAVD is divided into an initiation and a propagation phase (Fig. 1) [3]. The initiation phase starts by damage and stimulation of the VECs, which can be initiated by oxidative or mechanical stress [113, 152]. As bicuspid and unicuspid valves are subject to more mechanical stress, they often develop aortic valve stenosis one to two decades earlier [136]. The valvular damage alters the permeability and allows for infiltration of circulating lipoproteins, such as lipoprotein (a) (Lp(a)) and low-density lipoprotein (LDL) and immune cells, including monocytes and T lymphocytes [109, 111, 113]. Oxidized LDL (oxLDL) and Lp(a) stimulate and activate VICs and VECs, creating an inflammatory environment [111, 113] which further propagates the infiltration of immune cells [55]. The inflammatory milieu promotes VECs, VICs and macrophages to secrete extracellular vesicles, and induces apoptosis of macrophages and VICs, which release apoptotic bodies [72, 93]. Both processes cause microcalcifications by dystrophic calcification. Moreover, VICs are stimulated to differentiate into a myofibroblastic phenotype, causing VECM remodelling and fibrosis [25]. Further differentiation of myofibroblasts into an osteoblast-like phenotype results in biomineralization [58].

The propagation phase is characterized by accelerated fibrosis and calcification [102]. Accumulation of calcium and leaflet stiffening creates more mechanical stress and calcium deposition. A self-perpetuating cycle is established which eventually leads to narrowing of the valvular orifice [102, 177]. The valvular obstruction creates left ventricle systolic pressure overload, leading to myocardial hypertrophy, interstitial fibrosis and ultimately results in heart failure [82]. For the purpose of this review, we refer to excellent recent reviews for a more detailed general overview of the pathophysiology of CAVD [57, 72, 82, 115].

## CAVD and ASCVD, two sides of the same coin?

**Fig. 2:**
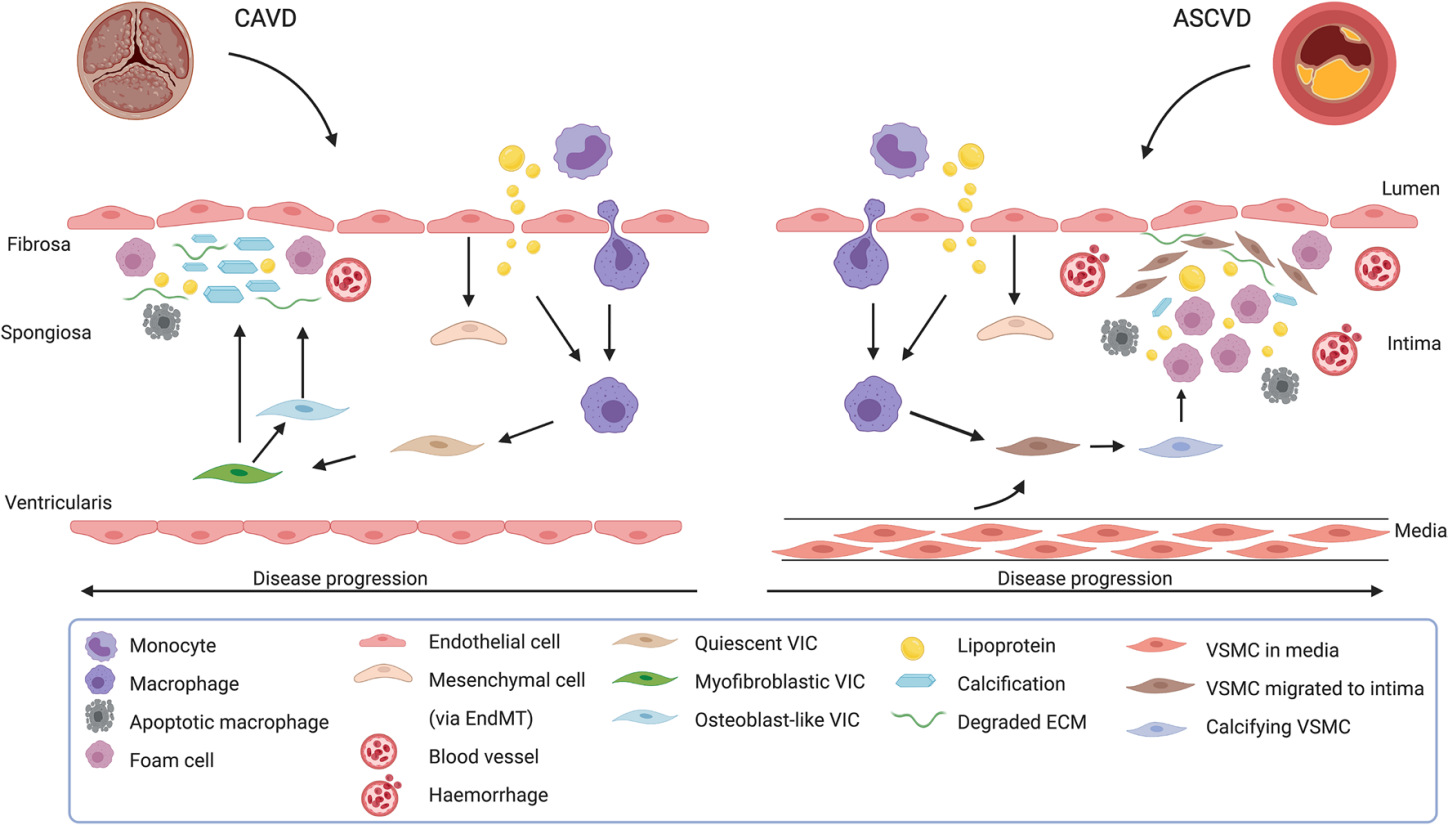
Schematic overview of innate immune cells in the pathophysiology of calcific aortic valve disease and atherosclerotic cardiovascular disease. The underlying pathophysiology of calcific aortic valve disease (CAVD) and atherosclerotic cardiovascular disease (ASCVD) shows many similarities in the initiation phase. In both CAVD as ASCVD, endothelial cells are damaged and activated, leading to lipoprotein infiltration and immune cell recruitment. The macrophages take up lipoproteins, leading to activation with subsequent secretion of proinflammatory cytokines and proteolytic enzymes and foam cell formation. Activated endothelial cells differentiate into mesenchymal cells (endothelial to mesenchymal transition) and transmigrate to the valvular interstitium or intima of the vessel wall. When the CAVD and ASCVD progress, the lesions start to show more differences. In CAVD, the valvular interstitial cells (VIC) are stimulated to differentiate to myofibroblasts or osteoblast-like cells and promote fibrosis and calcification, respectively. There are few foam cells and there is only little neovascularization. Apoptotic macrophages, VICs and foam cells contribute to the calcification. In ASCVD, foam cells are abundant and found across the intima and there is intraplaque haemorrhage due to leaky neovessels. Vascular smooth muscle cells (VSMCs) migrate from the media to the intima and form a fibrous cap. The activated macrophages stimulate osteoblastic differentiation of VSMCs subsequently. Macrophages, foam cells and VSMCs can die in advanced lesions by apoptosis, generating a necrotic core. Calcification is caused by osteoblast-like cells and the deposition of microcalcifications, which are generated by apoptotic cells

The development of CAVD is increasingly considered to be an atherosclerosis-like process, especially in the initiation phase [38]. Both CAVD and ASCVD represent chronic inflammatory disorders, which involve initial endothelial damage and activation, lipid deposition, immune cell recruitment, inflammation, neoangiogenesis and calcification (Fig. 2) [38, 102]. Pathological studies show active remodelling processes regulated by inflammation in both ASCVD and CAVD [27]. Importantly, in atherosclerosis*-*prone apolipoprotein E-deficient (*ApoE*^−/−^) mice, atherosclerotic plaques develop first in the aortic valves and aortic root [54]. Furthermore, CAVD and CAD often co-exist [31, 74], are both slowly progressive conditions with precursor lesions that remain asymptomatic for some time and they share important risk factors, including increased age, male sex, cigarette smoking, hypertension, kidney disease, diabetes mellitus, obesity, hyperlipidaemia, elevated Lp(a) levels and shared genetic susceptibility loci [20, 27, 130, 138, 151].

The role of the innate immune system is well established in the pathophysiology of ASCVD. Monocyte-derived macrophages are the principal immune cell type in atherosclerotic plaques and are involved in its initiation, progression and destabilization [95]. Limiting the influx of circulating monocytes into the arterial wall in atherosclerosis-prone mice prevents atherosclerotic plaque formation [17]. In these *ApoE*^−/−^ mice, the lesion size was reduced particularly in the valve leaflet region, where wild-type mice developed the most severe lesions [54]. Furthermore, targeting inflammation can prevent clinical atherosclerotic complications [139]. In addition, accumulating evidence points to the fact that circulating monocytes are characterized by an activated inflammatory phenotype in patients with established ASCVD or risk factors for ASCVD, including elevated LDL-cholesterol and Lp(a). Thus, activation of the innate immune system not only occurs in the inflammatory micro-environment of the plaque, but also in circulating monocytes [13, 14, 134, 163]. Moreover, recent studies have pointed out that the activation of innate immune cells in ASCVD also occur at the level of the myeloid progenitors in the bone marrow compartment [107, 164].

There are also important differences between CAVD and ASCVD, particularly in the advanced stages of the diseases. Firstly, there are patients with severe CAVD who do not suffer from advanced ASCVD and vice versa [62]. Secondly, statins do not prevent cardiovascular events in CAVD as opposed to their beneficial effect in patients with ASCVD, suggesting different pathophysiological processes [23, 48, 126]. Moreover, advanced lesions display histological differences, such as the fibrous cap and necrotic cores rich in foam cells in atheromas, which are not present in CAVD [89], and the limited presence of foam cells in calcified valves [71]. Lastly, adverse events in atherosclerosis are often related to plaque ruptures leading to acute coronary syndrome, while in CAVD, they are mostly caused by slowly progressive valve narrowing driven by progressive calcification [38, 90]. Despite the differences between CAVD and ASCVD, the profound commonalities in risk factors and similarities in (early) pathological features suggest overlap in pathophysiology, including a key role for inflammation and activation of the innate immune system. A systematic overview of the similarities and differences between CAVD and ASCD is given in Table 1.

**Table 1:**
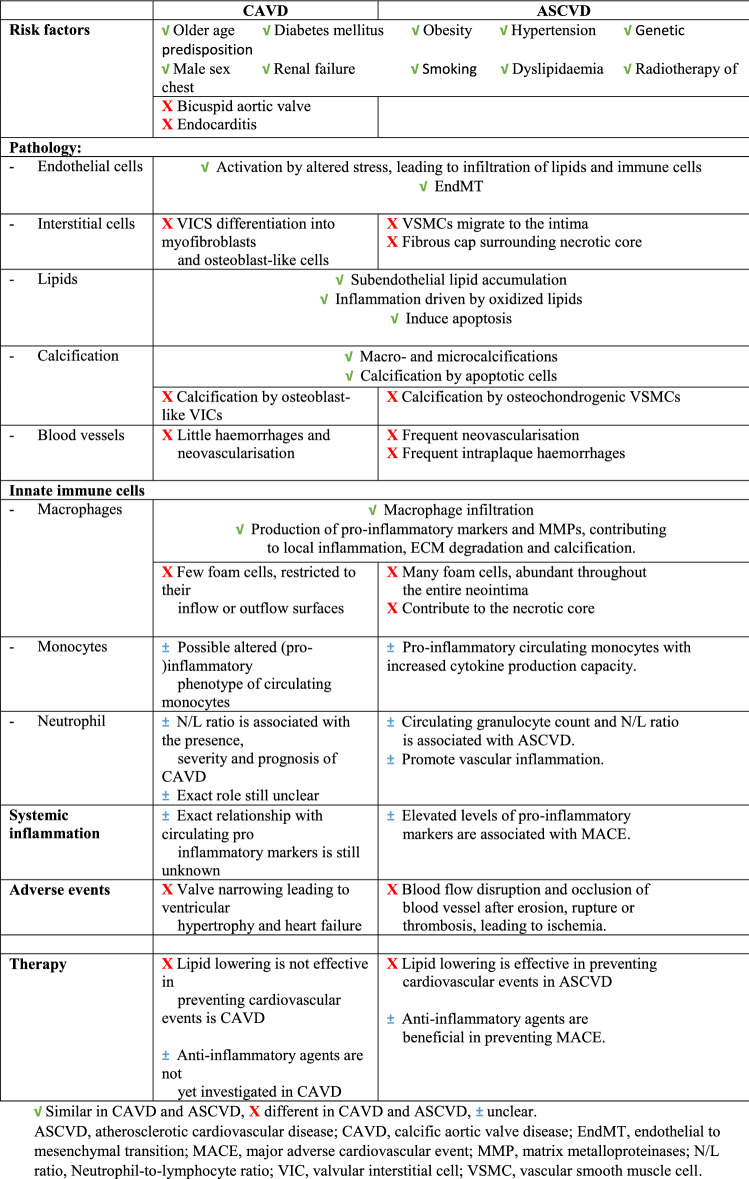
Similarities and differences between calcific aortic valve disease and atherosclerotic cardiovascular disease

## CAVD, a chronic inflammatory disease

Inflammation in CAVD occurs on several levels. Besides local inflammation in the aortic valves, inflammation can be observed in the circulation, by activated immune cells and increased inflammatory proteins. In this section, we will describe the various components of the inflammatory process in CAVD, from local to systemic inflammation, and compare this to the situation in ASCVD.

## Local valvular inflammation

CAVD develops by an active inflammatory process driven by infiltrated lipoproteins and immune cells. Histopathological examination of human calcified aortic valves shows subendothelial thickening with lipid deposition, immune cell infiltration (predominantly macrophages and T lymphocytes) and mineralization in early CAVD lesions and more advanced lesions in further progressed CAVD [29, 89, 94, 111, 113]. The inflammatory infiltrates are associated with valvular remodelling, neovascularization and osseous metaplasia [29]. Moreover, calcified aortic valves show an upregulated expression of multiple proinflammatory cytokines in total valve tissue, including interleukin (IL)-1β, IL-6, tumour necrosis factor (TNF), anti-inflammatory cytokines, as IL-10, and transforming growth factor (TGF)-β, as well as chemokines such as chemokine C-X-C ligand (CXCL) 5, CXCL9, chemokine C–C ligand (CCL) 19 and CCL 21 as summarized in Raddatz et al*.* [118]. The expression of the anti-inflammatory cytokine IL-37 is downregulated in calcified aortic valves [172]. The cells contributing to this valvular inflammation will be discussed below.

### Valvular endothelial damage

Early in CAVD development, altered haemodynamic forces on the valve affect the phenotype of VECs, leading to endothelial dysfunction. These altered forces result for example from hypertension [83], by stiffening of the valvular tissue due to ageing [143, 165], or from increased oxidative stress. In addition, other risk factors, such as diabetes or dyslipidaemia could precipitate endothelial dysfunction. The relevance of VEC injury is demonstrated by histopathological studies showing lipoprotein accumulation mainly in regions of low shear stress [109]. In the aortic valve, the damaged VECs subsequently express adhesion molecules, stimulating the recruitment of immune cells [55]. These immune cells produce cytokines leading to further stimulation of VECs and the transition of VECs into VICs by endothelial to mesenchymal transition [84]. Furthermore, VECs express endothelial nitric oxide synthase (eNOS), which regulates the production of nitric oxide. Calcified valves express reduced levels of eNOS, leading to increased oxidative stress which contributes to valvular inflammation by increasing lipoprotein oxidation [120, 153]. By these very processes, VECs promote inflammation, fibrosis and calcification.

This is comparable to the initiation phase in atherogenesis, where endothelial cell (EC) activation occurs in areas of disturbed shear stress, such as near arterial bifurcations, which permits circulating ApoB-containing lipoprotein and immune cells to enter the intimal space [81]. Stimulated ECs can also undergo endothelial to mesenchymal transition and migrate to the intima, where they can contribute to inflammation and intimal thickening [81]. Endothelial to mesenchymal transition is associated with atherosclerotic plaque instability [42].

### Valvular interstitial cells contribute to valvular inflammation

VICs are the most abundant cells in the valvular tissue and transiently transition into myofibroblasts during normal hemodynamic stress on the valvular tissue to remodel the ECM. During CAVD progression, the transition into myofibroblasts is more persistent, leading to pathological fibrosis. Ultimately, the myofibroblasts can differentiate into an osteoblast-like phenotype, which promotes calcium depositions. Although the exact underlying process driving valvular fibrosis and calcification remains uncertain [77], it is clear that proinflammatory communication between VICs and immune cells plays an important role. Firstly, Toll-like receptors (TLRs), especially TLR-2 and -4, are upregulated in VICs in calcified aortic valves. Several pathogen-associated molecular patterns (PAMPs) and damage-associated molecular patterns (DAMPs) promote inflammation and osteogenesis by activating the nuclear factor-κB (NF-κB) pathway in VICs via TLR stimulation. This leads to the production of proinflammatory and pro-calcifying molecules [52]. Recently, it was demonstrated that the upregulation of TLR2 in VICs is enhanced through paracrine signalling of TNF by activated monocytes [174]. IL-37, which shows a lower expression in calcified valves, suppresses the production of proinflammatory mediators by human VICs after TLR stimulation [172, 173]. Secondly, the differentiation of VICs into myofibroblasts or osteoblast-like cells is stimulated by cytokines, including TGF-β1 [76] or TNF, IFN-γ, IL-6 and receptor activator of NF-κB ligand (RANKL) [51, 57, 65]. In addition, myofibroblasts upregulate the expression of leptin-like oxidized LDL receptor-1 and scavenger CD36 receptors, leading to uptake of oxidized lipids and production of inflammatory molecules, resembling the foam cell-forming potential of macrophages in atherosclerotic lesions [147]. Lastly, VICs promote calcification via apoptosis and osteogenesis [77].

The processes of valvular fibrosis and calcification by VICs differ substantially from the extracellular matrix remodelling that occurs in atherosclerotic plaques, in which local inflammation stimulates smooth muscle cells to form a fibrous cap that shields to growing necrotic core, and stimulates vascular calcification which is summarized in detail in other reviews [37, 81].

### Macrophages

Macrophages are present in healthy valves, although histopathologic examination of explanted calcified valves demonstrates a higher abundance with CAVD progression [79, 113]. The majority of macrophages are located close to calcium deposits and areas of vascularization [99] and the inflammatory infiltrates are associated with active VECM remodelling, the severity of the stenosis and haemodynamic progression [29].

Macrophages are presumed essential in the initiation phase of CAVD (Fig. 1). After infiltration of circulating monocytes and differentiation into macrophages, the cells take up modified lipids via their scavenger receptors and can become foam cells [71, 111]. Calcified aortic valves contain only few foam cells, although fatty streaks are prominently found at the inflow and outflow surface of the valves [71]. In addition, macrophages are activated by cytokines and oxidized lipoproteins via pattern recognition receptors (PRRs) resulting in activation of the NF-κB pathway. Activated macrophages secrete multiple proinflammatory molecules, including IL-1β, IL-6, TNF, TGF-β, cathepsins, osteopontin and matrix metalloproteinases (MMPs) [47, 72]. The predominant macrophage subset found in human explanted calcified aortic valves consists of proinflammatory CD11c-positive macrophages [79] with an increased mRNA expression of iNOS, monocyte chemoattractant protein 1 (MCP-1), TNF, IL-6 and IL-12 [78]. In addition, the number of anti-inflammatory macrophages (CD206 +) is lower compared to healthy valves [79]. Chronic inflammation arises as the macrophages stimulate VECs, VICs and other immune cells, thereby promoting further immune cell recruitment, apoptosis, myofibroblastic and osteogenic differentiation of VICs and the differentiation of VECs into VICs via endothelial to mesenchymal transition [57, 96].

In the propagation phase, macrophages contribute to accelerated valvular fibrosis and calcification [57, 72]. Fibrous VECM remodelling is dependent on fibrosis and proteolysis. Activated macrophages produce TGF-β1, which in turn induces myofibroblastic differentiation of VICs [76] and secrete matrix remodelling proteins, such as MMPs, which promotes proteolysis.

Valvular calcification relies on two distinct processes; dystrophic calcification and biomineralization. Dystrophic calcification is defined by continuous deposition of microcalcifications and hydroxyapatite by apoptotic macrophages and VICs and extracellular vesicles, and is responsible for most of the calcification [72, 93]. Biomineralization is induced by osteoblast-like VICs, resembling osteogenesis. Inflammatory communication might play an important role in the calcification potential of these VICs [58, 79]. Conditioned medium of proinflammatory macrophages deactivates myofibroblasts and stimulates their proliferation, which is attributed to TNF and IL-1β [58]. TNF and IL-6, also secreted by proinflammatory macrophages, stimulate osteogenic differentiation of VICs and upregulate the expression of osteogenic markers by VICs [58, 79]. Macrophages can also contribute to clearance of mineralization through osteoclastogenesis after stimulation by RANKL and macrophage colony-stimulating factor (M-CSF), produced by osteoblast-like cells and T lymphocytes [18, 33]. These osteoclast-like cells can be found in calcified aortic valves and express proteins involved in mineral uptake and bone resorption [93, 97]. However, interferon (IFN)-γ produced by activated T lymphocytes impairs this osteoclastic activity. As a result, the osteoclast-like cells cannot counterbalance the osteoblastic activity from the VICs [98]. These processes, orchestrated by macrophages, progressively increase valvular fibrosis and calcification. The role of the adaptive immune system in the development of CAVD is discussed in recent excellent reviews [10, 118].

The role of macrophages in ASCVD is well established and shows many similarities with the pathogenesis of CAVD (Table 1). Monocyte-derived macrophages are the main immune cell type found in the atherosclerotic plaque and play a central role in all stages of atherogenesis [95]. Proinflammatory stimuli within the atherosclerotic plaque stimulate the macrophages to produce multiple proinflammatory chemokines and cytokines, creating an inflammatory milieu [24, 145]. However, contrary to in CAVD, foam cells are abundant in the atherosclerotic plaque and are distributed randomly across the neointima [71]. During progression of the atherosclerotic plaque, the death of foam cells and macrophages and subsequent impaired clearance of apoptotic cells by phagocytic cells (efferocytosis), contribute to the formation of a lipid and necrotic core [69, 80]. Moreover, macrophages contribute to destabilization of the atherosclerotic plaque by producing proteases [103].

### Oxidized lipids: central regulators of inflammation in CAVD

Lipoproteins take centre stage in the development and progression of CAVD by orchestrating the underlying inflammatory process. Observational studies and Mendelian randomization studies indicate that elevated LDL-cholesterol and Lp(a) levels are risk factors for CAVD [5, 7, 91, 137, 144]. Elevated Lp(a) levels are also associated with accelerated disease progression [7, 20, 176]. A single nucleotide polymorphism in the Lp(a) locus (rs1045872) is associated with elevated Lp(a) levels [7] and with aortic valve calcification [150]. Patients with mild to moderate CAVD with elevated Lp(a) levels suffer from a faster CAVD progression [21]. Lipids and lipid loaded macrophages localize predominantly in the subendothelial region of the fibrosa side of the valve [71, 111]. Valves containing higher amounts of oxLDL have denser inflammatory infiltrates, increased valvular tissue remodelling and higher expression of TNF [94]. The crucial role of lipoproteins is further demonstrated in the Reserva mouse model in which rapid normalisation of circulating cholesterol after a period of hyperlipidemia leads to a normalization of valvular oxidative stress, suppression of pro-osteogenic signalling, and a prevention of disease progression [91].

Lp(a) and oxLDL are powerful stimuli that drive valvular inflammation and calcification by oxidized phospholipids (OxPL) (Fig. 1). Lp(a) is the major carrier of OxPL in the circulation[163]. Antigen-presentation of oxLDL and apoB can activate T lymphocytes that subsequently stimulate VICs [92]. Oxidized lipids directly stimulate VECs leading to increased expression of bone morphogenic protein (BMP) 2 and adhesion molecules, thereby promoting calcification and the recruitment of immune cells [55, 109, 146]. OxLDL can augment the osteogenic response of human VICs through modulation of the NF-κB pathway and NOTCH1 activation [171]. Furthermore, OxPLs are transformed into lysophosphatidylcholine (LysoPC) and subsequently into lysophosphatidic acid (LPA) by Lp-PLA_2_ (lipoprotein-phospholipase A_2_), leading to apoptosis in VICs. LPA triggers an NF-κB-regulated inflammatory cascade in VICs and leads to increased expression of BMP2, IL-6 and Runt-related transcription factor 2 (Runx2) and secretion of alkaline phosphatase, stimulating valvular calcification [108, 130]. Additionally, LysoPC induces apoptosis in VICs [85]. In addition to Lp(a) and oxLDL, also triglyceride-rich lipoproteins are associated with an increased CAVD risk. Triglyceride-rich lipoproteins likely contribute to the lipid deposition and local inflammation by the release of monoacylglycerols and free fatty acids [66].

OxPL play a similar role in the development of ASCVD. OxLDL induces endothelial dysfunction and activation, triggers the recruitment of circulating immune cells and foam cell formation, and stimulates the VSMC migration and proliferation in the atherosclerotic plaque. Furthermore, oxLDL contributes to the destabilization of the atherosclerotic plaque by inducing apoptosis and the release of MMPs [116]. The OxPL on Lp(a) cause a proinflammatory monocyte response and inflammation of the arterial wall in humans [163].

## Beyond the valve: systemic inflammation and circulating immune cell activation

There is accumulating evidence that CAVD, just like ASCVD, is characterized not only by local valvular inflammation, but also by low-grade systemic inflammation, although this is still controversial. In ASCVD, it is well established that elevated circulating levels of proinflammatory markers, e.g. IL-6 and high-sensitive C-reactive protein (hsCRP), are associated with major adverse cardiovascular events, which is independent of other traditional risk factors [68, 121, 124, 169]. Some studies also suggest an association between circulating hsCRP and the presence, severity and progression of CAVD [50, 88, 129], mostly in patients with advanced CAVD. Other studies, however, did not find a relationship between elevated hsCRP, the presence of aortic sclerosis or CAVD and disease progression [59, 106]. Also, no correlation was found between hsCRP and valvular inflammatory infiltrates [88]. The exact relationship between hsCRP levels and CAVD therefore remains uncertain. In a small observational study, patients with severe CAVD had increased levels of circulating TNF, but it is still not known whether this is causally related to CAVD pathophysiology or results from the haemodynamic consequences of CAVD [67]. More evidence points to activation of circulating innate immune cells in CAVD pathophysiology, which is highlighted below.

### Monocyte activation in CAVD

Human monocytes can be divided in three subsets based on the surface expression of CD14 and CD16: classical monocytes are CD14++ CD16−, intermediate monocytes CD14++ CD16 + and non-classical monocytes are CD14+ CD16++ [141, 178]. In addition, other surface markers can be used to characterize subsets with specific functions. For example, classical monocytes are known for their high C–C chemokine receptor type 2 (CCR2) expression, whereas intermediate monocytes have high CCR5 expression [46, 141]. In general, circulating CD16+ monocytes, especially the intermediate monocytes, are associated with atherosclerotic disease [125, 155].

Few studies have characterized monocyte subsets in the setting of CAVD. Most of these studies are cross-sectional and observational in small patient groups, hence results need to be interpreted with caution. Shimoni et al*.* showed increased levels of CD14+ monocytes in patients with severe CAVD compared to controls which was inversely correlated with the aortic valve area surface [133]. Hewing et al*.* showed that patients with severe CAVD have higher levels of proinflammatory intermediate monocytes [59]. The level of intermediate monocytes has been reported to drop after aortic valve replacement (AVR) [60, 101], although the effect of the surgical intervention itself was not evaluated with a control group. The relationship between disease severity and monocyte subtypes is still unclear [59, 101]. It remains speculative whether the increased levels of circulating (intermediate) monocytes play a causal role in the pathophysiology of CAVD or, are rather a consequence of the disease through haemodynamic changes or valvular inflammation. Although these studies suggest that the phenotype of circulating monocytes is altered in patients with CAVD, more in-depth exploration of monocyte function and phenotype has not yet been performed.

Changes in monocyte phenotype have been described in the setting of ASCVD and also for several risk factors for CAVD. In patients with established CAD, circulating monocytes are characterized by an augmented cytokine production capacity [14, 134]. In addition, patients with elevated levels of Lp(a) show an increased level of intermediate monocytes, which correlates with OxPL/apoB, independent of circulating CRP and IL-6 [73]. Also, monocytes from patients with increased levels of LDL-cholesterol as well as Lp(a) show a hyperresponsive state, with an enhanced cytokine production capacity and increased transendothelial migration [13, 131, 163], which is associated with increased arterial wall inflammation in high Lp(a) conditions [163]. Circulating monocytes stem from bone marrow hematopoietic stem and progenitor cells. In patients with CAD, the bone marrow myeloid progenitor cells are programmed towards a proinflammatory phenotype [107]. This has never been investigated in the context of CAVD.

### Neutrophils: important players in CAVD?

The role of neutrophils in cardiovascular inflammation and their possible contribution to the pathogenesis of CAVD only recently gained attention. Patients with severe CAVD have a higher absolute circulating neutrophil count compared to healthy controls [140]. An increased neutrophil-to-lymphocyte ratio is associated with the presence, severity and prognosis of CAVD [9, 26, 140]. In addition, Kopytek et al*.* demonstrated that calcified aortic valves exhibit significantly more neutrophil extracellular trap (NET) formation compared to healthy valves and that the amount of valvular NETs correlates with disease severity, suggesting a role for neutrophils in the progression of CAVD [70]. In contrast, by using electron microscopy, Kostyunin et al*.* did not find neutrophils to be present in severely calcified aortic valves [71]. More research is needed to identify the exact role of neutrophils in the fibro-calcific remodelling of the aortic valve.

The role of neutrophils and NETs in ASCVD is more established. A recent prospective epidemiological study demonstrated that circulating granulocyte count is strongly associated with the future occurrence of ASCVD [43]. Furthermore, an increased granulocyte-to-lymphocyte ratio is a risk factor for ASCVD [43]. Neutrophil activation and recruitment is promoted by chemokines, such as CC-chemokine ligand 5, during atherogenesis [135]. Activated neutrophils then secrete granule proteins, including Cathepsin G, at the luminal side, which can activate chemokines resulting in further myeloid cell recruitment. The secretion of ROS and myeloperoxidase, which mediates LDL oxidation and subsequently promotes foam cell formation, further promotes atherosclerotic disease progression [135]. Besides, neutrophils can promote vascular wall inflammation by the secretion of proinflammatory microvesicles [56] and the formation of NETs [35].

## Proposed new mechanisms for circulating immune cell activation in CAVD

**Fig. 3:**
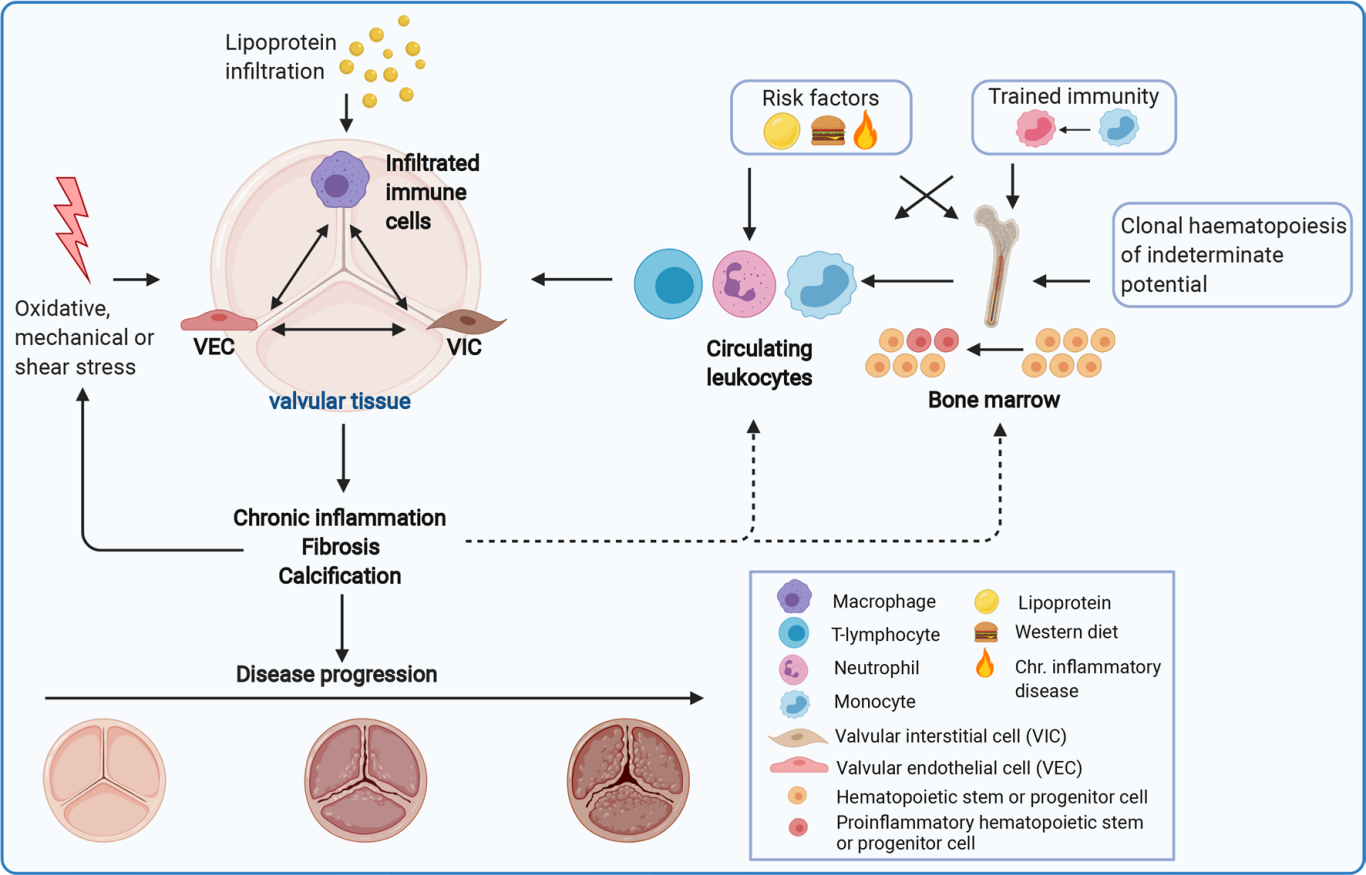
A schematic illustration of how systemic immune cell reprogramming can contribute to CAVD pathophysiology. Oxidative, mechanical or shear stress damages and activates valvular endothelial cells (VECs), altering endothelial permeability. This causes lipoproteins and immune cells to infiltrate the valvular tissue, creating an inflammatory environment. Local migrated immune cells and activated VECs and valvular interstitial cells (VICs) continue to stimulate each other, thereby causing chronic inflammation, fibrosis and calcification. This leads to valve leaflet stiffening and thickening, which increases mechanical stress, establishing a self-perpetuating cycle. Activation of innate immune cells, such as monocytes, macrophages and neutrophils, contributes to the initiation and development of CAVD. Risk factors for CAVD, such as hyperlipidaemia, elevated Lp(a) levels and a Western diet, activate hematopoietic stem and progenitor cells (HSPCs) and circulating immune cells. Trained immunity can lead to a persistent pro-inflammatory phenotype of circulating innate immune cells and myeloid progenitor cells. Clonal haematopoiesis of indeterminate potential (CHIP) results in a pro-inflammatory phenotype of HSPCs. The proinflammatory leukocytes infiltrate the valvular tissue and contribute to the development of CAVD by creating an inflammatory environment. The chronic inflammation that arises might in turn impact on HSPCs and circulating leukocytes

Until now, it is unclear how activation of innate immune cells might contribute to CAVD pathophysiology. Trained immunity and CHIP are recently described immunological mechanism that could potentially contribute to long-term activation of innate immune cells and we propose that these mechanisms could contribute to the development of ASCVD and CAVD (Fig. 3).

### Trained immunity

Trained immunity describes the phenomenon that innate immune cells, including monocytes and macrophages, are able to adapt their function after a first encounter with a DAMP or PAMP, leading to a long-term hyperresponsive phenotype [11]. Although this mechanism is beneficial in the context of recurrent infections, it might be detrimental in chronic inflammatory diseases in which these innate immune cells themselves contribute to pathophysiology and tissue damage, such as atherosclerosis [45]. Trained immunity is dependent on intracellular metabolic and epigenetic reprogramming, resulting in a persistent proinflammatory phenotype, characterized by an increased cytokine production capacity [100]. In vitro, brief exposure of isolated human monocytes to oxLDL, Lp(a), uric acid or adrenaline/noradrenaline induces a trained macrophage phenotype [12, 161, 163].

Recent studies translated the concept of trained immunity to patients with established ASCVD or risk factors for ASCVD. In patients with CAD, the augmented cytokine production of circulating monocytes was associated with increased glycolysis and enrichment of epigenetic histone markers, characteristic of trained immunity [14]. In addition, monocytes from treatment-naïve patients with familial hypercholesterolaemia have an increased cytokine production capacity and enrichment of activating histone modifications on the promotors of these cytokine genes, which persisted for three months after cholesterol lowering with statins [13]. A similar hyperresponsive monocyte phenotype was observed in patients with elevated Lp(a) levels [163].

The prolonged presence of monocytes with a trained hyperresponsive phenotype is explained by the fact that training occurs at the level of myeloid precursors in the bone marrow [100]. In this regard, it was recently shown that isolated bone marrow mononuclear cells of patients with severe CAD demonstrate an increased cytokine production capacity and a higher metabolic rate than individuals without CAD. The bone marrow composition of the CAD patients showed skewing towards myelopoiesis and the hematopoietic stem and progenitor cells demonstrated enriched monocyte and neutrophil related pathways [107]. Moreover, several risk factors for both CAVD and ASCVD are described to reprogram myeloid progenitor cells in mouse models [28, 132, 167]. A Western type diet in *Ldlr*^*−*/−^ mice induces long-term epigenetic and transcriptomic reprogramming of myeloid progenitor cells, leading to increased myelopoiesis and augmented innate immune responses, which persist despite switching back to a chow diet [28]. Furthermore, bone marrow transplantation from Western type diet fed *Ldlr*^*−/−*^ mice into *Ldlr*^*−/−*^ mice on a chow diet was associated with an increased number of circulating inflammatory leukocytes and an increased aortic root plaque sizes compared to chow fed donor bone marrow [167]. Similarly, transplantation of bone marrow progenitors from diabetic mice into normoglycemic atherosclerosis-prone mice accelerated atherosclerosis by trained immunity [41].

### Clonal haematopoiesis and CAVD

Recently, clonal haematopoiesis of indeterminate potential (CHIP) has been identified as an important mechanism of innate immune cell activation [49, 64]. Clonal haematopoiesis (CH) describes the disproportionate clonal growth of leukocytes arising from a single progenitor cell harbouring a somatic mutation, without the presence of haematologic malignancy [142]. CH is rare in young patients, but the prevalence increases with age, affecting > 10% of individuals older than 65 years [53, 170]. CH-driver mutations (CHDM) provide a survival advantage to the mutated cells and allow progressive clonal expansion, leading to accumulation of circulating mutant leucocytes [142]. CHDM occur mainly in genes encoding for epigenetic regulators, such as ten–eleven translocation 2 (TET2) and DNA methyltransferase 3A (DNMT3A). CH is associated with an augmented all-cause mortality risk [53, 63] and an increased risk of atherosclerotic CVD [49, 64]. At least for TET2 driver mutations, this association appears to be driven by a hyper-inflammatory phenotype of clonal monocytes. This is mediated, at least partly, due to the fact that TET2-deficient macrophages exhibit an increased NLRP3 inflammasome-dependent secretion of IL-1β [49], which is key to the development of atherosclerosis [123]. DNMT3A deficiency has been associated with diminished immunosuppressive function of myeloid-derived suppressor cells, proinflammatory activation of mast cells and an increased production of IFN-γ by T lymphocytes. However, a direct pathophysiological connection between DNMT3A loss-of-function and atherosclerosis has not yet been established [6]. Interestingly, it was demonstrated that increased haematological stem cell proliferation, driven by atherosclerosis itself, can accelerate CH, creating a vicious cycle [61].

Recently, in patients with severe CAVD undergoing transcatheter AVR, a higher prevalence of DNMT3A and TET2 mutations was found in circulating monocytes compared to other cohorts of healthy subjects or to subjects with CAD. Patients with CHDM had a markedly increased all-cause mortality during the first eight months after a successful transcatheter AVR. Compared to non-CHDM carriers, patients with TET2 mutations had elevated levels of proinflammatory non-classical monocytes and patients with DNMT3A mutations showed proinflammatory T lymphocyte polarization [87]. Moreover, another study demonstrated that monocytes of patients with severe degenerative aortic valve stenosis or chronic postischemic heart failure, who harbour DNMT3A or TET2 CHDM, appeared to be primed for excessive inflammatory responses by assessing the transcriptome of circulating peripheral monocytes of CAVD by single-cell RNA sequencing [2].

## Imaging of valvular inflammation and calcification

Currently, echocardiography is used to clinically assess the severity of the aortic valve stenosis and computed tomography (CT) is used to quantify valvular macrocalcification and disease severity and progression [36]. However, it is important that we can properly measure inflammation, both systemically and locally, to obtain a better understanding of how inflammation contributes to CAVD pathophysiology. Active arterial wall inflammation can be visualized by ^18^F-fluorodeoxyglucose (^18^F-FDG) positron-emission tomography (PET) combined with CT [127]. In atherosclerotic plaques, FDG-uptake correlates with plaque macrophage burden [149]. Marincheva-Sancheva et al*.* were the first to demonstrate higher valvular ^18^F-FDG uptake in patients with mild and moderate CAVD, but not in patients with severe CAVD, compared to controls. This suggests that inflammation plays a more important role in the early phases of CAVD than in advanced disease. Also, patients with higher baseline ^18^F-FDG uptake showed an increased disease progression [86]. In addition, ^18^F-sodium fluoride (^18^F-NaF) PET–CT can be used, to detect recent calcification activity and calcium remodelling. Dweck et al*.* demonstrated that both valvular ^18^F-FDG and ^18^F-NaF uptake were higher in CAVD patients compared to controls. However, the ^18^F-NaF uptake displayed a more progressive rise with disease severity than the more modest increased uptake of ^18^F-FDG. Moreover, uptake of ^18^F-NaF and not ^18^F-FDG strongly correlates with disease severity [39]. A follow-up study demonstrated that both ^18^F-FDG and ^18^F-NaF uptake independently predict disease progression and adverse outcomes. ^18^F-NaF uptake correlates strongly with CT calcium score progression and novel calcium depositions develop in the same distribution as baseline ^18^F-NaF uptake. Interestingly, Abdelbacky et al*.* demonstrated that valvular ^18^F-FDG uptake independently predicted subsequent calcification in patients without CAVD at baseline and thus indicated that inflammation precedes calcification [1]. Together, these studies support that valvular inflammation plays an important role in early CAVD and precedes calcification, which predominantly drives disease progression in later stages. These findings correspond to recent findings in patients with atherosclerosis, showing mainly ^18^F-FDG uptake in arterial segments without advanced plaques, suggesting an arterial inflammatory state at early stages of atherosclerosis [44].

Finally, another emerging imaging technique in the imaging of CAVD patients is the use of PET combined with magnetic resonance imaging (MRI). In addition to the potential to detect valvular inflammation with tracers, the PET/MRI has particular added value in assessing prognostic factors, including characterization of the myocardial tissue for remodelling, fibrosis and hypertrophy [156].

## Pharmacological treatment to prevent severe CAVD

Although CAVD can be diagnosed at an early stage, there is currently no effective medical treatment available and ‘watchful waiting’ is the only option until endovascular or surgical intervention is needed. Elucidation of the pathophysiology of CAVD will hopefully reveal potential pharmacological targets for prevention or treatment. Given the pivotal roles of (oxidized) lipoproteins, various trials have been performed with lipid-lowering drugs (Table 2). In addition, targeting systemic inflammation and the immune system might be an effective strategy to prevent or treat CAVD. For other examined agents in the search for a pharmacological treatment for CAVD, we refer to the recent review of Donato et al*.* [34].

**Table 2:** Major published clinical trials investigating lipid-lowering therapy in calcific aortic valve disease

Trial	Patients	Study design	Intervention	Primary + echocardiographic outcomes	Inflammatory outcome	Refs.
SALTIRE	155 patients with CAVD and aortic jet velocity ≥ 2.5 m/s	Double-blind, placebo-controlled RCT FU: 25 months (median, range 7–36)	Atorvastatin 80 mg per day versus placebo	- Aortic jet velocity change/year Atorvastatin: 0.199 ± 0.210 m/s Placebo:0.203 ± 0.208 m/s (*P* = 0.95) - Calcification progression/year Atorvastatin: 22.3 ± 21.0% Placebo: 21.7 ± 19.8% (*P* = 0.93)	None mentioned	[30]
TASS	47 patients with asymptomatic CAVD with mean systolic gradient ≥ 15 mmHg and peak velocity ≥ 2.0 m/s	Placebo-controlled RCT FU: 2.3 ± 1.2 years (median, SD)	Atorvastatin 20 mg per day versus placebo	- HR 0.78 (95% CI 0.32–1.87; *P* = 0.569) for MACE - CAVD progression: Mean gradient at last FU (mmHg): Atorvastatin: 31.3 ± 12.3 Placebo: 29.9 ± 14.8 (P = NS) Aortic valve calcification at last FU (Agatston score): Atorvastatin: 2979 ± 1228 Placebo: 2749 ± 1376 (P = NS)	CRP level (mg/dl) 0.14 ± 0.66 in atorvastatin group versus 0.33 ± 1.39 in control group (*P* < 0.05) after 12 month FU. P = NS after 24 months FU	[32]
SEAS	1873 patients with mild to moderate, asymptomatic CAVD	Multicentre, double-blind, placebo-controlled RCT FU: 52.2 months (median)	Simvastatin 40 mg + ezetimibe 10 mg per day versus placebo	- HR 0.96 (95% CI 0.83 to 1.12; P = 0.59) for MACE - Increase in peak velocity (m/s): Simvastatin + ezetimibe: 0.61 ± 0.59 Placebo: 0.62 ± 0.61 (P = 0.83) - Increase mean gradient (mmHg/year): Simvastatin + ezetimibe: 2.7 ± 0.1 Placebo: 2.8 ± 0.1 - Decrease in AVA (cm2/year): − 0.03 ± 0.01 in both groups	None mentioned	[126]
ASTRON-OMER	269 patients with mild or moderate CAVD	Multicentre, double-blind, placebo-controlled RCT FU: 3.5 years (median, IQR 2.1–4.5)	Rosuvastatin 40 mg per day versus placebo	- Annualized peak AS gradient increase (mmHg): Rosuvastatin: 6.3 ± 6.9 Placebo: 6.1 ± 8.2 (*P* = 0.83) - Increase in mean gradient (mmHg): Rosuvastatin: 10.7 Placebo: 9.6 (*P* = 0.49) - Decrease in AVA (cm2): Rosuvastatin: − 0.19 Placebo: − 0.16 (*P* = 0.79)	CRP reduction of 0.33 mg/L in rosuvastatin group compared to an increase of 0.095 mg/L in the placebo group (*P* = 0.002)	[23]
FOURIER (post hoc analysis)	27,564 patients with stable atherosclerotic disease receiving statin therapy	Multicentre, double-blind, placebo-controlled RCT FU: 26 months (median, IQR 22–30)	Subc. evolocumab injection (140 mg biweekly or 420 mg monthly) versus placebo	- Post hoc analysis: HR 0.48 (95% CI, 0.25–0.93) for overall CAVD events after first year of evolocumab	None mentioned	[15]

*AVA* aortic valve area, *CAVD* calcific aortic valve disease, *CI* confidential interval, *CRP* C-reactive protein, *FU* follow-up; HR, hazard ratio, *IQR* interquartile range, *LDL* low-density lipoprotein, *Lp(a)* lipoprotein (a), *MACE* major adverse cardiovascular events, *RCT* randomized controlled trial, *subc.* subcutaneous

### Lipid-lowering therapy

The pivotal role of oxidized lipoproteins in CAVD suggest lipid-lowering as possible treatment. However, four double-blind randomized controlled trials (RCTs) showed that statins do not halt or slow down CAVD progression, in contrast to strong beneficial effects on ASCVD (Table 2) [23, 30, 32, 126]. This might be explained by the fact that these patients already had established CAVD with a self-perpetuating calcification process. Another explanation is that statins do not lower Lp(a), and can even increase Lp(a) levels [154]. Additionally, statins are suggested to increase vascular calcifications, which might increase plaque stability and reduce the number of cardiovascular events in the context of atherosclerosis [117, 122], but further drives calcification and subsequent disease progression in CAVD. Interestingly, a post hoc meta-analysis of three studies investigating the effect of 80 mg atorvastatin mg per day in patients with stable CVD without CAVD, did not show a reduced risk for developing CAVD [8]. However, two of the three included trials compared low-dose statin therapy as control; treatment naïve patients as control group may have resulted in different results.

Convertase subtilisin/kexin type 9 (PCSK9) inhibitors are monoclonal antibodies that bind to circulating PCSK9 and inhibit PCSK9-mediated LDL-receptor degradation. This results in a powerful LDL-cholesterol lowering. In addition, PCSK9 inhibitors also lower Lp(a) concentration by 20–30% respectively [110, 128]. The FOURIER trial examined the effect of the PCSK9-inhibitor evolocumab in patients with stable atherosclerotic disease receiving statin therapy and demonstrated a reduced cardiovascular event risk [128]. Interestingly, a post hoc analysis of this trial displayed that treatment with evolocumab might also reduce CAVD related events [15], which is in line with the findings that patients with a PCSK9 loss-of-function mutation have a reduced CAVD risk [75]. These findings suggest that Lp(a) lowering might be able to prevent or slow down the progression of CAVD. These preliminary findings need further validation with RCTs and currently, the effects of PCSK9 inhibitors in CAVD are being investigated (Table 3) [157]. In addition, the effect of Lp(a) lowering on CAVD progression by niacin is being explored (Table 3) [158].

**Table 3:** Clinical trials investigating lipid-lowering therapies of interest for calcific aortic valve disease

Trial	Patients	Study design	Intervention	Primary outcome	Clinicaltrials. gov identifier	Refs.
PCSK9 inhibitors in the progression of aortic stenosis	140 patients with mild to moderate CAVD	Phase 2 multicentre randomized, double-blind, placebo-controlled clinical trial Follow-up 2 years	Subcutaneous injection of EPC biweekly versus placebo	Calcium score progression measured by cardiac CT (Agatston score) and by NaF PET–CT	NCT03051360	[157]
EAVaLL	238 patients with aortic sclerosis or mild CAVD and elevated Lp(a)	Phase 2, randomized, placebo-controlled clinical trial Follow-up 2 years	Niacin 1500–2000 mg versus placebo	Calcium score progression measured by cardiac CT	NCT02109614	[158]
Lp(a) HORIZON	7680 patients with established CVD and elevated Lp(a)	Phase 3 multicentre, randomized, double-blind, placebo-controlled clinical trial Follow-up 4 years	Subcutaneous injection of TQQJ230 80 mg monthly versus placebo	Time to first occurrence of MACE	NCT04023552	[160]

*CAVD* calcific aortic valve disease, *CI* confidential interval, *CT* computed tomography, *CVD* cardiovascular disease, *HR* hazard ratio, *IQR* interquartile range, *LDL* low-density lipoprotein, *Lp(a)* lipoprotein (a), *MACE* major adverse cardiovascular events, *NaF* sodium fluoride, *PET* positron emission tomography

Another promising therapy targeting Lp(a) is antisense oligonucleotide therapy. These synthetic oligonucleotides bind to apoB or apo(a) mRNA in hepatocytes, resulting in a decreased production of apoB-containing lipoproteins and Lp(a). This leads to a significant reduction in circulating OxPL and Lp(a) [168]. Currently, the HORIZON trial, a large phase 3 multicentre RCT is recruiting patients to assess the impact of the antisense oligonucleotide TQJ230 on major cardiovascular events in patients with CVD (Table 3) [160]. This therapy might also be beneficial in patients with CAVD.

### Anti-inflammatory agents

To the best of our knowledge, there are no clinical trials investigating the effect of anti-inflammatory drugs on CAVD. In the setting of ASCVD, however, recently several RCTs have reported effectiveness of the anti-inflammatory drugs colchicine and canakinumab in the prevention of CVD, following the publication of many neutral trials with other anti-inflammatory drugs [175]. Given the overlap in inflammatory components in the pathophysiology of atherosclerotic CVD and CAVD, these drugs might also have beneficial effects in the context of CAVD.

The low-dose colchicine for secondary prevention of cardiovascular disease (LoDoCo) trial demonstrated that colchicine, a broad anti-inflammatory agent, reduces the risk of cardiovascular events in patients with stable CAD [104]. This beneficial effect was confirmed in two large RCTs in patients after recent myocardial infarction [148], or with chronic coronary disease [105]. In addition to its known inhibitory effect on inflammasome activation, colchicine appears to attenuate neutrophil activation [112]. Given these actions, colchicine might be an attractive candidate to limit CAVD progression and this hypothesis will soon be tested in a new randomized controlled clinical trial [159].

The Canakinumab Anti-inflammatory Thrombosis Outcome Study (CANTOS) demonstrated that inhibition with the human anti-IL-1β antibody canakinumab decreases cardiovascular event rates in patients with a recent myocardial infarction [123]. This trial did not evaluate the effects on CAVD. Nonetheless, IL-1β production by macrophages induces calcification by vascular mesenchymal cells [22], which makes IL-1β also an interesting candidate for CAVD treatment. Furthermore, the CHIP-associated aberrant inflammation, such as the IL-1β overexpression by TET2 deficient macrophages, further strengthens the possible role for IL-1β as therapeutic target in CAVD [49]. As a consequence of the promising results of the CANTOS trial, attention has moved upstream of the IL-1β pathway to target inflammasomes and downstream IL-6. Animal studies using NLRP3 inflammasome inhibitors and IL-6 receptor antagonists have shown promising results in targeting atherosclerosis [4, 162, 166], but larger trials are needed to investigate their clinical relevance.

## Conclusion

In conclusion, evidence is gradually accumulating that the complex pathophysiology of CAVD is an inflammatory process in which various immune cells plays a prominent role. Considering the central role of innate immune cells in the pathophysiology of ASCVD and the similarities between ASCVD and CAVD, it is rational to hypothesize that activation of the innate immune system also contributes to the initiation and progression of CAVD. Further elucidation of the driving processes of innate immune cell activation in CAVD, including trained immunity and CHIP, might identify novel therapeutic targets that can be used for prevention and treatment of CAVD. The recent exciting evidence that anti-inflammatory strategies potently limits atherosclerotic CVD further underscores the importance of this scientific field.

## References

[1] Abdelbaky A, Corsini E, Figueroa AL, Subramanian S, Fontanez S, Emami H, Hoffmann U, Narula J, Tawakol A (2015). Early aortic valve inflammation precedes calcification: a longitudinal FDG-PET/CT study. Atherosclerosis.

[2] Abplanalp WT, Mas-Peiro S, Cremer S, John D, Dimmeler S, Zeiher AM (2020). Association of clonal hematopoiesis of indeterminate potential with inflammatory gene expression in patients with severe degenerative aortic valve stenosis or chronic postischemic heart failure. JAMA Cardiol.

[3] Aikawa E, Nahrendorf M, Sosnovik D, Lok VM, Jaffer FA, Aikawa M, Weissleder R (2007). Multimodality molecular imaging identifies proteolytic and osteogenic activities in early aortic valve disease. Circulation.

[4] Akita K, Isoda K, Sato-Okabayashi Y, Kadoguchi T, Kitamura K, Ohtomo F, Shimada K, Daida H (2017). An interleukin-6 receptor antibody suppresses atherosclerosis in atherogenic mice. Front Cardiovasc Med.

[5] Allara E, Morani G, Carter P, Gkatzionis A, Zuber V, Foley CN, Rees JMB, Mason AM, Bell S, Gill D, Lindström S, Butterworth AS, Di Angelantonio E, Peters J, Burgess S (2019). Genetic determinants of lipids and cardiovascular disease outcomes: a wide-angled mendelian randomization investigation. Circ Genom Precis Med.

[6] Amoros-Perez M, Fuster JJ (2020). Clonal hematopoiesis driven by somatic mutations: a new player in atherosclerotic cardiovascular disease. Atherosclerosis.

[7] Arsenault BJ, Boekholdt SM, Dube MP, Rheaume E, Wareham NJ, Khaw KT, Sandhu MS, Tardif JC (2014). Lipoprotein(a) levels, genotype, and incident aortic valve stenosis: a prospective Mendelian randomization study and replication in a case-control cohort. Circ Cardiovasc Genet.

[8] Arsenault BJ, Boekholdt SM, Mora S, DeMicco DA, Bao W, Tardif JC, Amarenco P, Pedersen T, Barter P, Waters DD (2014). Impact of high-dose atorvastatin therapy and clinical risk factors on incident aortic valve stenosis in patients with cardiovascular disease (from TNT, IDEAL, and SPARCL). Am J Cardiol.

[9] Avci A, Elnur A, Goksel A, Serdar F, Servet I, Atilla K, Mustafa TM, Cuneyt T, Yeliz G, Mustafa B, Metin EA (2014). The relationship between neutrophil/lymphocyte ratio and calcific aortic stenosis. Echocardiography.

[10] Bartoli-Leonard F, Zimmer J, Aikawa E (2021). Innate and adaptative immunity: the understudied driving force of heart valve disease. Cardiovasc Res.

[11] Bekkering S, Domínguez-Andrés J, Joosten LAB, Riksen NP, Netea MG (2021). Trained immunity: reprogramming innate immunity in health and disease. Annu Rev Immunol.

[12] Bekkering S, Quintin J, Joosten LA, van der Meer JW, Netea MG, Riksen NP (2014). Oxidized low-density lipoprotein induces long-term proinflammatory cytokine production and foam cell formation via epigenetic reprogramming of monocytes. Arterioscler Thromb Vasc Biol.

[13] Bekkering S, Stiekema LCA, Bernelot Moens S, Verweij SL, Novakovic B, Prange K, Versloot M, Roeters van Lennep JE, Stunnenberg H, de Winther M, Stroes ESG, Joosten LAB, Netea MG, Riksen NP (2019). Treatment with statins does not revert trained immunity in patients with familial hypercholesterolemia. Cell Metab.

[14] Bekkering S, van den Munckhof I, Nielen T, Lamfers E, Dinarello C, Rutten J, de Graaf J, Joosten LA, Netea MG, Gomes ME, Riksen NP (2016). Innate immune cell activation and epigenetic remodeling in symptomatic and asymptomatic atherosclerosis in humans in vivo. Atherosclerosis.

[15] Bergmark BA, O'Donoghue ML, Murphy SA, Kuder JF, Ezhov MV, Ceška R, Gouni-Berthold I, Jensen HK, Tokgozoglu SL, Mach F, Huber K, Gaciong Z, Lewis BS, Schiele F, Jukema JW, Pedersen TR, Giugliano RP, Sabatine MS (2020). An Exploratory analysis of proprotein convertase subtilisin/kexin type 9 inhibition and aortic stenosis in the FOURIER trial. JAMA Cardiol.

[16] Bonow RO, Greenland P (2015). Population-wide trends in aortic stenosis incidence and outcomes. Circulation.

[17] Boring L, Gosling J, Cleary M, Charo IF (1998). Decreased lesion formation in CCR2-/- mice reveals a role for chemokines in the initiation of atherosclerosis. Nature.

[18] Boyle WJ, Simonet WS, Lacey DL (2003). Osteoclast differentiation and activation. Nature.

[19] Butcher JT, Mahler GJ, Hockaday LA (2011). Aortic valve disease and treatment: the need for naturally engineered solutions. Adv Drug Deliv Rev.

[20] Capoulade R, Chan KL, Yeang C, Mathieu P, Bosse Y, Dumesnil JG, Tam JW, Teo KK, Mahmut A, Yang X, Witztum JL, Arsenault BJ, Despres JP, Pibarot P, Tsimikas S (2015). Oxidized phospholipids, lipoprotein(a), and progression of calcific aortic valve stenosis. J Am Coll Cardiol.

[21] Capoulade R, Yeang C, Chan KL, Pibarot P, Tsimikas S (2018). Association of mild to moderate aortic valve stenosis progression with higher lipoprotein(a) and oxidized phospholipid levels: secondary analysis of a randomized clinical trial. JAMA Cardiol.

[22] Ceneri N, Zhao L, Young BD, Healy A, Coskun S, Vasavada H, Yarovinsky TO, Ike K, Pardi R, Qin L, Qin L, Tellides G, Hirschi K, Meadows J, Soufer R, Chun HJ, Sadeghi MM, Bender JR, Morrison AR (2017). Rac2 modulates atherosclerotic calcification by regulating macrophage interleukin-1β production. Arterioscler Thromb Vasc Biol.

[23] Chan KL, Teo K, Dumesnil JG, Ni A, Tam J (2010). Effect of Lipid lowering with rosuvastatin on progression of aortic stenosis: results of the aortic stenosis progression observation: measuring effects of rosuvastatin (ASTRONOMER) trial. Circulation.

[24] Chávez-Sánchez L, Madrid-Miller A, Chávez-Rueda K, Legorreta-Haquet MV, Tesoro-Cruz E, Blanco-Favela F (2010). Activation of TLR2 and TLR4 by minimally modified low-density lipoprotein in human macrophages and monocytes triggers the inflammatory response. Hum Immunol.

[25] Chen JH, Yip CY, Sone ED, Simmons CA (2009). Identification and characterization of aortic valve mesenchymal progenitor cells with robust osteogenic calcification potential. Am J Pathol.

[26] Cho KI, Cho SH, Her AY, Singh GB, Shin ES (2016). Prognostic utility of neutrophil-to-lymphocyte ratio on adverse clinical outcomes in patients with severe calcific aortic stenosis. PLoS ONE.

[27] Cho KI, Sakuma I, Sohn IS, Jo SH, Koh KK (2018). Inflammatory and metabolic mechanisms underlying the calcific aortic valve disease. Atherosclerosis.

[28] Christ A, Gunther P, Lauterbach MAR, Duewell P, Biswas D, Pelka K, Scholz CJ, Oosting M, Haendler K, Bassler K, Klee K, Schulte-Schrepping J, Ulas T, Moorlag S, Kumar V, Park MH, Joosten LAB, Groh LA, Riksen NP, Espevik T, Schlitzer A, Li Y, Fitzgerald ML, Netea MG, Schultze JL, Latz E (2018). Western diet triggers NLRP3-dependent innate immune reprogramming. Cell.

[29] Cote N, Mahmut A, Bosse Y, Couture C, Page S, Trahan S, Boulanger MC, Fournier D, Pibarot P, Mathieu P (2013). Inflammation is associated with the remodeling of calcific aortic valve disease. Inflammation.

[30] Cowell SJ, Newby DE, Prescott RJ, Bloomfield P, Reid J, Northridge DB, Boon NA (2005). A randomized trial of intensive lipid-lowering therapy in calcific aortic stenosis. N Engl J Med.

[31] D'Ascenzo F, Conrotto F, Giordana F, Moretti C, D'Amico M, Salizzoni S, Omedè P, La Torre M, Thomas M, Khawaja Z, Hildick-Smith D, Ussia G, Barbanti M, Tamburino C, Webb J, Schnabel RB, Seiffert M, Wilde S, Treede H, Gasparetto V, Napodano M, Tarantini G, Presbitero P, Mennuni M, Rossi ML, Gasparini M, Biondi Zoccai G, Lupo M, Rinaldi M, Gaita F, Marra S (2013). Mid-term prognostic value of coronary artery disease in patients undergoing transcatheter aortic valve implantation: a meta-analysis of adjusted observational results. Int J Cardiol.

[32] Dichtl W, Alber HF, Feuchtner GM, Hintringer F, Reinthaler M, Bartel T, Süssenbacher A, Grander W, Ulmer H, Pachinger O, Müller S (2008). Prognosis and risk factors in patients with asymptomatic aortic stenosis and their modulation by atorvastatin (20 mg). Am J Cardiol.

[33] Doherty TM, Asotra K, Fitzpatrick LA, Qiao JH, Wilkin DJ, Detrano RC, Dunstan CR, Shah PK, Rajavashisth TB (2003). Calcification in atherosclerosis: bone biology and chronic inflammation at the arterial crossroads. Proc Natl Acad Sci U S A.

[34] Donato M, Ferri N, Lupo MG, Faggin E, Rattazzi M (2020). Current evidence and future perspectives on pharmacological treatment of calcific aortic valve stenosis. Int J Mol Sci.

[35] Doring Y, Libby P, Soehnlein O (2020). Neutrophil extracellular traps participate in cardiovascular diseases: recent experimental and clinical insights. Circ Res.

[36] Doris MK, Everett RJ, Shun-Shin M, Clavel MA, Dweck MR (2019). The role of imaging in measuring disease progression and assessing novel therapies in aortic stenosis. JACC Cardiovasc Imaging.

[37] Durham AL, Speer MY, Scatena M, Giachelli CM, Shanahan CM (2018). Role of smooth muscle cells in vascular calcification: implications in atherosclerosis and arterial stiffness. Cardiovasc Res.

[38] Dweck MR, Boon NA, Newby DE (2012). Calcific aortic stenosis: a disease of the valve and the myocardium. J Am Coll Cardiol.

[39] Dweck MR, Jones C, Joshi NV, Fletcher AM, Richardson H, White A, Marsden M, Pessotto R, Clark JC, Wallace WA, Salter DM, McKillop G, van Beek EJ, Boon NA, Rudd JH, Newby DE (2012). Assessment of valvular calcification and inflammation by positron emission tomography in patients with aortic stenosis. Circulation.

[40] Edep ME, Shirani J, Wolf P, Brown DL (2000). Matrix metalloproteinase expression in nonrheumatic aortic stenosis. Cardiovasc Pathol.

[41] Edgar L, Akbar N, Braithwaite AT, Krausgruber T, Gallart-Ayala H, Bailey J, Corbin AL, Khoyratty TE, Chai JT, Alkhalil M, Rendeiro AF, Ziberna K, Arya R, Cahill TJ, Bock C, Laurencikiene J, Crabtree MJ, Lemieux ME, Riksen NP, Netea MG, Wheelock CE, Channon KM, Rydén M, Udalova IA, Carnicer R, Choudhury RP (2021). Hyperglycemia induces trained immunity in macrophages and their precursors and promotes atherosclerosis. Circulation.

[42] Evrard SM, Lecce L, Michelis KC, Nomura-Kitabayashi A, Pandey G, Purushothaman KR, d'Escamard V, Li JR, Hadri L, Fujitani K, Moreno PR, Benard L, Rimmele P, Cohain A, Mecham B, Randolph GJ, Nabel EG, Hajjar R, Fuster V, Boehm M, Kovacic JC (2016). Endothelial to mesenchymal transition is common in atherosclerotic lesions and is associated with plaque instability. Nat Commun.

[43] Fani L, van der Willik KD, Bos D, Leening MJG, Koudstaal PJ, Rizopoulos D, Ruiter R, Stricker BHC, Kavousi M, Ikram MA, Ikram MK (2020). The association of innate and adaptive immunity, subclinical atherosclerosis, and cardiovascular disease in the Rotterdam Study: a prospective cohort study. PLoS Med.

[44] Fernández-Friera L, Fuster V, López-Melgar B, Oliva B, Sánchez-González J, Macías A, Pérez-Asenjo B, Zamudio D, Alonso-Farto JC, España S, Mendiguren J, Bueno H, García-Ruiz JM, Ibañez B, Fernández-Ortiz A, Sanz J (2019). Vascular inflammation in subclinical atherosclerosis detected by hybrid PET/MRI. J Am Coll Cardiol.

[45] Flores-Gomez D, Bekkering S, Netea MG, Riksen NP (2021). Trained immunity in atherosclerotic cardiovascular disease. Arterioscler Thromb Vasc Biol.

[46] Franca CN, Izar MCO, Hortencio MNS, do Amaral JB, Ferreira CES, Tuleta ID, Fonseca FAH (2017). Monocyte subtypes and the CCR2 chemokine receptor in cardiovascular disease. Clin Sci (Lond).

[47] Freeman RV, Otto CM (2005). Spectrum of calcific aortic valve disease: pathogenesis, disease progression, and treatment strategies. Circulation.

[48] Fulcher J, O'Connell R, Voysey M, Emberson J, Blackwell L, Mihaylova B, Simes J, Collins R, Kirby A, Colhoun H, Braunwald E, La Rosa J, Pedersen TR, Tonkin A, Davis B, Sleight P, Franzosi MG, Baigent C, Keech A (2015). Efficacy and safety of LDL-lowering therapy among men and women: meta-analysis of individual data from 174,000 participants in 27 randomised trials. Lancet.

[49] Fuster JJ, MacLauchlan S, Zuriaga MA, Polackal MN, Ostriker AC, Chakraborty R, Wu CL, Sano S, Muralidharan S, Rius C, Vuong J, Jacob S, Muralidhar V, Robertson AA, Cooper MA, Andres V, Hirschi KK, Martin KA, Walsh K (2017). Clonal hematopoiesis associated with TET2 deficiency accelerates atherosclerosis development in mice. Science.

[50] Galante A, Pietroiusti A, Vellini M, Piccolo P, Possati G, De Bonis M, Grillo RL, Fontana C, Favalli C (2001). C-reactive protein is increased in patients with degenerative aortic valvular stenosis. J Am Coll Cardiol.

[51] Galeone A, Brunetti G, Oranger A, Greco G, Di Benedetto A, Mori G, Colucci S, Zallone A, Paparella D, Grano M (2013). Aortic valvular interstitial cells apoptosis and calcification are mediated by TNF-related apoptosis-inducing ligand. Int J Cardiol.

[52] Garcia-Rodriguez C, Parra-Izquierdo I, Castanos-Mollor I, Lopez J, San Roman JA, Sanchez Crespo M (2018). Toll-like receptors, inflammation, and calcific aortic valve disease. Front Physiol.

[53] Genovese G, Kahler AK, Handsaker RE, Lindberg J, Rose SA, Bakhoum SF, Chambert K, Mick E, Neale BM, Fromer M, Purcell SM, Svantesson O, Landen M, Hoglund M, Lehmann S, Gabriel SB, Moran JL, Lander ES, Sullivan PF, Sklar P, Gronberg H, Hultman CM, McCarroll SA (2014). Clonal hematopoiesis and blood-cancer risk inferred from blood DNA sequence. N Engl J Med.

[54] Getz GS, Reardon CA (2012). Animal models of atherosclerosis. Arterioscler Thromb Vasc Biol.

[55] Ghaisas NK, Foley JB, O'Briain DS, Crean P, Kelleher D, Walsh M (2000). Adhesion molecules in nonrheumatic aortic valve disease: endothelial expression, serum levels and effects of valve replacement. J Am Coll Cardiol.

[56] Gomez I, Ward B, Souilhol C, Recarti C, Ariaans M, Johnston J, Burnett A, Mahmoud M, Luong LA, West L, Long M, Parry S, Woods R, Hulston C, Benedikter B, Niespolo C, Bazaz R, Francis S, Kiss-Toth E, van Zandvoort M, Schober A, Hellewell P, Evans PC, Ridger V (2020). Neutrophil microvesicles drive atherosclerosis by delivering miR-155 to atheroprone endothelium. Nat Commun.

[57] Goody PR, Hosen MR, Christmann D, Niepmann ST, Zietzer A, Adam M, Bönner F, Zimmer S, Nickenig G, Jansen F (2020). Aortic valve stenosis: from basic mechanisms to novel therapeutic targets. Arterioscler Thromb Vasc Biol.

[58] Grim JC, Aguado BA, Vogt BJ, Batan D, Andrichik CL, Schroeder ME, Gonzalez-Rodriguez A, Yavitt FM, Weiss RM, Anseth KS (2020). Secreted factors from proinflammatory macrophaged promote an osteoblast-like phenotype in valvular interstitial cells. Arterioscler Thromb Vasc Biol.

[59] Hewing B, Au SC, Ludwig A, Ellerbroek R, van Dijck P, Hartmann L, Grubitzsch H, Giannini C, Laule M, Stangl V, Baumann G, Stangl K (2017). Severe aortic valve stenosis in adults is associated with increased levels of circulating intermediate monocytes. J Cardiovasc Transl Res.

[60] Hewing B, Ellerbroek R, Au SC, Stangl V, Dreger H, Laule M, Grubitzsch H, Knebel F, Baumann G, Ludwig A, Stangl K (2017). Levels of circulating intermediate monocytes decrease after aortic valve replacement in patients with severe aortic stenosis. Thromb Haemost.

[61] Heyde A, Rohde D, McAlpine CS, Zhang S, Hoyer FF, Gerold JM, Cheek D, Iwamoto Y, Schloss MJ, Vandoorne K, Iborra-Egea O, Muñoz-Guijosa C, Bayes-Genis A, Reiter JG, Craig M, Swirski FK, Nahrendorf M, Nowak MA, Naxerova K (2021). Increased stem cell proliferation in atherosclerosis accelerates clonal hematopoiesis. Cell.

[62] Jackson V, Eriksson MJ, Caidahl K, Eriksson P, Franco-Cereceda A (2014). Ascending aortic dilatation is rarely associated with coronary artery disease regardless of aortic valve morphology. J Thorac Cardiovasc Surg.

[63] Jaiswal S, Fontanillas P, Flannick J, Manning A, Grauman PV, Mar BG, Lindsley RC, Mermel CH, Burtt N, Chavez A, Higgins JM, Moltchanov V, Kuo FC, Kluk MJ, Henderson B, Kinnunen L, Koistinen HA, Ladenvall C, Getz G, Correa A, Banahan BF, Gabriel S, Kathiresan S, Stringham HM, McCarthy MI, Boehnke M, Tuomilehto J, Haiman C, Groop L, Atzmon G, Wilson JG, Neuberg D, Altshuler D, Ebert BL (2014). Age-related clonal hematopoiesis associated with adverse outcomes. N Engl J Med.

[64] Jaiswal S, Natarajan P, Silver AJ, Gibson CJ, Bick AG, Shvartz E, McConkey M, Gupta N, Gabriel S, Ardissino D, Baber U, Mehran R, Fuster V, Danesh J, Frossard P, Saleheen D, Melander O, Sukhova GK, Neuberg D, Libby P, Kathiresan S, Ebert BL (2017). Clonal hematopoiesis and risk of atherosclerotic cardiovascular disease. N Engl J Med.

[65] Kaden JJ, Bickelhaupt S, Grobholz R, Haase KK, Sarikoç A, Kiliç R, Brueckmann M, Lang S, Zahn I, Vahl C, Hagl S, Dempfle CE, Borggrefe M (2004). Receptor activator of nuclear factor kappaB ligand and osteoprotegerin regulate aortic valve calcification. J Mol Cell Cardiol.

[66] Kaltoft M, Langsted A, Nordestgaard BG (2020). Triglycerides and remnant cholesterol associated with risk of aortic valve stenosis: Mendelian randomization in the Copenhagen General Population Study. Eur Heart J.

[67] Kapadia SR, Yakoob K, Nader S, Thomas JD, Mann DL, Griffin BP (2000). Elevated circulating levels of serum tumor necrosis factor-alpha in patients with hemodynamically significant pressure and volume overload. J Am Coll Cardiol.

[68] Kaptoge S, Di Angelantonio E, Lowe G, Pepys MB, Thompson SG, Collins R, Danesh J (2010). C-reactive protein concentration and risk of coronary heart disease, stroke, and mortality: an individual participant meta-analysis. Lancet.

[69] Kavurma MM, Rayner KJ, Karunakaran D (2017). The walking dead: macrophage inflammation and death in atherosclerosis. Curr Opin Lipidol.

[70] Kopytek M, Kolasa-Trela R, Zabczyk M, Undas A, Natorska J (2019). NETosis is associated with the severity of aortic stenosis: links with inflammation. Int J Cardiol.

[71] Kostyunin A, Mukhamadiyarov R, Glushkova T, Bogdanov L, Shishkova D, Osyaev N, Ovcharenko E, Kutikhin A (2020). Ultrastructural pathology of atherosclerosis, calcific aortic valve disease, and bioprosthetic heart valve degeneration: commonalities and differences. Int J Mol Sci.

[72] Kostyunin AE, Yuzhalin AE, Ovcharenko EA, Kutikhin AG (2019). Development of calcific aortic valve disease: do we know enough for new clinical trials?. J Mol Cell Cardiol.

[73] Krychtiuk KA, Kastl SP, Hofbauer SL, Wonnerth A, Goliasch G, Ozsvar-Kozma M, Katsaros KM, Maurer G, Huber K, Dostal E, Binder CJ, Pfaffenberger S, Oravec S, Wojta J, Speidl WS (2015). Monocyte subset distribution in patients with stable atherosclerosis and elevated levels of lipoprotein(a). J Clin Lipidol.

[74] Kvidal P, Bergström R, Hörte LG, Ståhle E (2000). Observed and relative survival after aortic valve replacement. J Am Coll Cardiol.

[75] Langsted A, Nordestgaard BG, Benn M, Tybjærg-Hansen A, Kamstrup PR (2016). PCSK9 R46L loss-of-function mutation reduces lipoprotein(a), LDL cholesterol, and risk of aortic valve stenosis. J Clin Endocrinol Metab.

[76] Latif N, Quillon A, Sarathchandra P, McCormack A, Lozanoski A, Yacoub MH, Chester AH (2015). Modulation of human valve interstitial cell phenotype and function using a fibroblast growth factor 2 formulation. PLoS ONE.

[77] Lee SH, Choi JH (2016). Involvement of immune cell network in aortic valve stenosis: communication between valvular interstitial cells and immune cells. Immune Netw.

[78] Li C, Xu S, Gotlieb AI (2013). The progression of calcific aortic valve disease through injury, cell dysfunction, and disruptive biologic and physical force feedback loops. Cardiovasc Pathol.

[79] Li G, Qiao W, Zhang W, Li F, Shi J, Dong N (2017). The shift of macrophages toward M1 phenotype promotes aortic valvular calcification. J Thorac Cardiovasc Surg.

[80] Libby P (2021). The changing landscape of atherosclerosis. Nature.

[81] Libby P (2021). Inflammation during the life cycle of the atherosclerotic plaque. Cardiovasc Res.

[82] Lindman BR, Clavel MA, Mathieu P, Iung B, Lancellotti P, Otto CM, Pibarot P (2016). Calcific aortic stenosis. Nat Rev Dis Primers.

[83] Linefsky J, Katz R, Budoff M, Probstfield J, Owens D, Takasu J, Shavelle D, Ouyang P, Psaty B, O'Brien KD (2011). Stages of systemic hypertension and blood pressure as correlates of computed tomography-assessed aortic valve calcium (from the Multi-Ethnic Study of Atherosclerosis). Am J Cardiol.

[84] Mahler GJ, Farrar EJ, Butcher JT (2013). Inflammatory cytokines promote mesenchymal transformation in embryonic and adult valve endothelial cells. Arterioscler Thromb Vasc Biol.

[85] Mahmut A, Boulanger MC, El Husseini D, Fournier D, Bouchareb R, Despres JP, Pibarot P, Bosse Y, Mathieu P (2014). Elevated expression of lipoprotein-associated phospholipase A2 in calcific aortic valve disease: implications for valve mineralization. J Am Coll Cardiol.

[86] Marincheva-Savcheva G, Subramanian S, Qadir S, Figueroa A, Truong Q, Vijayakumar J, Brady TJ, Hoffmann U, Tawakol A (2011). Imaging of the aortic valve using fluorodeoxyglucose positron emission tomography increased valvular fluorodeoxyglucose uptake in aortic stenosis. J Am Coll Cardiol.

[87] Mas-Peiro S, Hoffmann J, Fichtlscherer S, Dorsheimer L, Rieger MA, Dimmeler S, Vasa-Nicotera M, Zeiher AM (2020). Clonal haematopoiesis in patients with degenerative aortic valve stenosis undergoing transcatheter aortic valve implantation. Eur Heart J.

[88] Mazzone A, Epistolato MC, De Caterina R, Storti S, Vittorini S, Sbrana S, Gianetti J, Bevilacqua S, Glauber M, Biagini A, Tanganelli P (2004). Neoangiogenesis, T-lymphocyte infiltration, and heat shock protein-60 are biological hallmarks of an immunomediated inflammatory process in end-stage calcified aortic valve stenosis. J Am Coll Cardiol.

[89] Mazzone A, Epistolato MC, Gianetti J, Castagnini M, Sassi C, Ceravolo R, Bevilacqua S, Glauber M, Biagini A, Tanganelli P (2006). Biological features (inflammation and neoangiogenesis) and atherosclerotic risk factors in carotid plaques and calcified aortic valve stenosis: two different sites of the same disease?. Am J Clin Pathol.

[90] Milin AC, Vorobiof G, Aksoy O, Ardehali R (2014). Insights into aortic sclerosis and its relationship with coronary artery disease. J Am Heart Assoc.

[91] Miller JD, Weiss RM, Serrano KM, Brooks RM, Berry CJ, Zimmerman K, Young SG, Heistad DD (2009). Lowering plasma cholesterol levels halts progression of aortic valve disease in mice. Circulation.

[92] Miteva K, Madonna R, De Caterina R, Van Linthout S (2018). Innate and adaptive immunity in atherosclerosis. Vascul Pharmacol.

[93] Mohler ER, Gannon F, Reynolds C, Zimmerman R, Keane MG, Kaplan FS (2001). Bone formation and inflammation in cardiac valves. Circulation.

[94] Mohty D, Pibarot P, Despres JP, Cote C, Arsenault B, Cartier A, Cosnay P, Couture C, Mathieu P (2008). Association between plasma LDL particle size, valvular accumulation of oxidized LDL, and inflammation in patients with aortic stenosis. Arterioscler Thromb Vasc Biol.

[95] Moore KJ, Sheedy FJ, Fisher EA (2013). Macrophages in atherosclerosis: a dynamic balance. Nat Rev Immunol.

[96] Nadlonek N, Lee JH, Reece TB, Weyant MJ, Cleveland JC, Meng X, Fullerton DA (2013). Interleukin-1 Beta induces an inflammatory phenotype in human aortic valve interstitial cells through nuclear factor kappa Beta. Ann Thorac Surg.

[97] Nagy E, Eriksson P, Yousry M, Caidahl K, Ingelsson E, Hansson GK, Franco-Cereceda A, Back M (2013). Valvular osteoclasts in calcification and aortic valve stenosis severity. Int J Cardiol.

[98] Nagy E, Lei Y, Martinez-Martinez E, Body SC, Schlotter F, Creager M, Assmann A, Khabbaz K, Libby P, Hansson GK, Aikawa E (2017). Interferon-gamma released by activated CD8(+) T lymphocytes impairs the calcium resorption potential of osteoclasts in calcified human aortic valves. Am J Pathol.

[99] Natorska J, Marek G, Sadowski J, Undas A (2016). Presence of B cells within aortic valves in patients with aortic stenosis: relation to severity of the disease. J Cardiol.

[100] Netea MG, Dominguez-Andres J, Barreiro LB, Chavakis T, Divangahi M, Fuchs E, Joosten LAB, van der Meer JWM, Mhlanga MM, Mulder WJM, Riksen NP, Schlitzer A, Schultze JL, Stabell Benn C, Sun JC, Xavier RJ, Latz E (2020). Defining trained immunity and its role in health and disease. Nat Rev Immunol.

[101] Neuser J, Galuppo P, Fraccarollo D, Willig J, Kempf T, Berliner D, Bauersachs J, Widder JD (2017). Intermediate CD14++CD16+ monocytes decline after transcatheter aortic valve replacement and correlate with functional capacity and left ventricular systolic function. PLoS ONE.

[102] New SE, Aikawa E (2011). Molecular imaging insights into early inflammatory stages of arterial and aortic valve calcification. Circ Res.

[103] Newby AC (2008). Metalloproteinase expression in monocytes and macrophages and its relationship to atherosclerotic plaque instability. Arterioscler Thromb Vasc Biol.

[104] Nidorf SM, Eikelboom JW, Budgeon CA, Thompson PL (2013). Low-dose colchicine for secondary prevention of cardiovascular disease. J Am Coll Cardiol.

[105] Nidorf SM, Fiolet ATL, Mosterd A, Eikelboom JW, Schut A, Opstal TSJ, The SHK, Xu XF, Ireland MA, Lenderink T, Latchem D, Hoogslag P, Jerzewski A, Nierop P, Whelan A, Hendriks R, Swart H, Schaap J, Kuijper AFM, van Hessen MWJ, Saklani P, Tan I, Thompson AG, Morton A, Judkins C, Bax WA, Dirksen M, Alings MMW, Hankey GJ, Budgeon CA, Tijssen JGP, Cornel JH, Thompson PL (2020). Colchicine in patients with chronic coronary disease. N Engl J Med.

[106] Novaro GM, Katz R, Aviles RJ, Gottdiener JS, Cushman M, Psaty BM, Otto CM, Griffin BP (2007). Clinical factors, but not C-reactive protein, predict progression of calcific aortic-valve disease: the Cardiovascular Health Study. J Am Coll Cardiol.

[107] Noz MP, Bekkering S, Groh L, Nielen TM, Lamfers EJ, Schlitzer A, El Messaoudi S, van Royen N, Huys EH, Preijers FW, Smeets EM, Aarntzen EH, Zhang B, Li Y, Bremmers ME, van der Velden WJ, Dolstra H, Joosten LA, Gomes ME, Netea MG, Riksen NP (2020). Reprogramming of bone marrow myeloid progenitor cells in patients with severe coronary artery disease. Elife.

[108] Nsaibia MJ, Boulanger MC, Bouchareb R, Mkannez G, Le Quang K, Hadji F, Argaud D, Dahou A, Bossé Y, Koschinsky ML, Pibarot P, Arsenault BJ, Marette A, Mathieu P (2017). OxLDL-derived lysophosphatidic acid promotes the progression of aortic valve stenosis through a LPAR1-RhoA-NF-κB pathway. Cardiovasc Res.

[109] O'Brien KD, Reichenbach DD, Marcovina SM, Kuusisto J, Alpers CE, Otto CM (1996). Apolipoproteins B, (a), and E accumulate in the morphologically early lesion of 'degenerative' valvular aortic stenosis. Arterioscler Thromb Vasc Biol.

[110] O'Donoghue ML, Fazio S, Giugliano RP, Stroes ESG, Kanevsky E, Gouni-Berthold I, Im K, Lira Pineda A, Wasserman SM, Češka R, Ezhov MV, Jukema JW, Jensen HK, Tokgözoğlu SL, Mach F, Huber K, Sever PS, Keech AC, Pedersen TR, Sabatine MS (2019). Lipoprotein(a), PCSK9 inhibition, and cardiovascular risk. Circulation.

[111] Olsson M, Thyberg J, Nilsson J (1999). Presence of oxidized low density lipoprotein in nonrheumatic stenotic aortic valves. Arterioscler Thromb Vasc Biol.

[112] Opstal TSJ, Hoogeveen RM, Fiolet ATL, Silvis MJM, The SHK, Bax WA, de Kleijn DPV, Mosterd A, Stroes ESG, Cornel JH (2020). Colchicine attenuates inflammation beyond the inflammasome in chronic coronary artery disease: a LoDoCo2 proteomic substudy. Circulation.

[113] Otto CM, Kuusisto J, Reichenbach DD, Gown AM, O'Brien KD (1994). Characterization of the early lesion of 'degenerative' valvular aortic stenosis. Histological and immunohistochemical studies. Circulation.

[114] Otto CM, Prendergast B (2014). Aortic-valve stenosis–from patients at risk to severe valve obstruction. N Engl J Med.

[115] Peeters F, Meex SJR, Dweck MR, Aikawa E, Crijns H, Schurgers LJ, Kietselaer B (2018). Calcific aortic valve stenosis: hard disease in the heart: a biomolecular approach towards diagnosis and treatment. Eur Heart J.

[116] Pirillo A, Norata GD, Catapano AL (2013). LOX-1, OxLDL, and atherosclerosis. Mediators Inflamm.

[117] Puri R, Nicholls SJ, Shao M, Kataoka Y, Uno K, Kapadia SR, Tuzcu EM, Nissen SE (2015). Impact of statins on serial coronary calcification during atheroma progression and regression. J Am Coll Cardiol.

[118] Raddatz MA, Madhur MS, Merryman WD (2019). Adaptive immune cells in calcific aortic valve disease. Am J Physiol Heart Circ Physiol.

[119] Rajamannan NM, Evans FJ, Aikawa E, Grande-Allen KJ, Demer LL, Heistad DD, Simmons CA, Masters KS, Mathieu P, O'Brien KD, Schoen FJ, Towler DA, Yoganathan AP, Otto CM (2011). Calcific aortic valve disease: not simply a degenerative process: a review and agenda for research from the National Heart and Lung and Blood Institute Aortic Stenosis Working Group. Executive summary: Calcific aortic valve disease-2011 update. Circulation.

[120] Richards J, El-Hamamsy I, Chen S, Sarang Z, Sarathchandra P, Yacoub MH, Chester AH, Butcher JT (2013). Side-specific endothelial-dependent regulation of aortic valve calcification: interplay of hemodynamics and nitric oxide signaling. Am J Pathol.

[121] Ridker PM, Cushman M, Stampfer MJ, Tracy RP, Hennekens CH (1997). Inflammation, aspirin, and the risk of cardiovascular disease in apparently healthy men. N Engl J Med.

[122] Ridker PM, Danielson E, Fonseca FA, Genest J, Gotto AM, Kastelein JJ, Koenig W, Libby P, Lorenzatti AJ, MacFadyen JG, Nordestgaard BG, Shepherd J, Willerson JT, Glynn RJ (2008). Rosuvastatin to prevent vascular events in men and women with elevated C-reactive protein. N Engl J Med.

[123] Ridker PM, Everett BM, Thuren T, MacFadyen JG, Chang WH, Ballantyne C, Fonseca F, Nicolau J, Koenig W, Anker SD, Kastelein JJP, Cornel JH, Pais P, Pella D, Genest J, Cifkova R, Lorenzatti A, Forster T, Kobalava Z, Vida-Simiti L, Flather M, Shimokawa H, Ogawa H, Dellborg M, Rossi PRF, Troquay RPT, Libby P, Glynn RJ, Group CT (2017). Antiinflammatory therapy with canakinumab for atherosclerotic disease. N Engl J Med.

[124] Ridker PM, Rifai N, Stampfer MJ, Hennekens CH (2000). Plasma concentration of interleukin-6 and the risk of future myocardial infarction among apparently healthy men. Circulation.

[125] Rogacev KS, Cremers B, Zawada AM, Seiler S, Binder N, Ege P, Grosse-Dunker G, Heisel I, Hornof F, Jeken J, Rebling NM, Ulrich C, Scheller B, Bohm M, Fliser D, Heine GH (2012). CD14++CD16+ monocytes independently predict cardiovascular events: a cohort study of 951 patients referred for elective coronary angiography. J Am Coll Cardiol.

[126] Rossebø AB, Pedersen TR, Boman K, Brudi P, Chambers JB, Egstrup K, Gerdts E, Gohlke-Bärwolf C, Holme I, Kesäniemi YA, Malbecq W, Nienaber CA, Ray S, Skjaerpe T, Wachtell K, Willenheimer R (2008). Intensive lipid lowering with simvastatin and ezetimibe in aortic stenosis. N Engl J Med.

[127] Rudd JH, Narula J, Strauss HW, Virmani R, Machac J, Klimas M, Tahara N, Fuster V, Warburton EA, Fayad ZA, Tawakol AA (2010). Imaging atherosclerotic plaque inflammation by fluorodeoxyglucose with positron emission tomography: ready for prime time?. J Am Coll Cardiol.

[128] Sabatine MS, Giugliano RP, Keech AC, Honarpour N, Wiviott SD, Murphy SA, Kuder JF, Wang H, Liu T, Wasserman SM, Sever PS, Pedersen TR, Committee FS, Investigators (2017). Evolocumab and Clinical outcomes in patients with cardiovascular disease. New England J Med.

[129] Sanchez PL, Santos JL, Kaski JC, Cruz I, Arribas A, Villacorta E, Cascon M, Palacios IF, Martin-Luengo C (2006). Relation of circulating C-reactive protein to progression of aortic valve stenosis. Am J Cardiol.

[130] Schnitzler JG, Ali L, Groenen AG, Kaiser Y, Kroon J (2019). Lipoprotein(a) as orchestrator of calcific aortic valve stenosis. Biomolecules.

[131] Schnitzler JG, Hoogeveen RM, Ali L, Prange KH, Waissi F, van Weeghel M, Bachmann JC, Versloot M, Borrelli MJ, Yeang C, de Kleijn D, Houtkooper RH, Koschinsky M, de Winther MP, Groen AK, Witztum JL, Tsimikas S, Stroes ES, Kroon J (2020). Atherogenic lipoprotein(a) increases vascular glycolysis, thereby facilitating inflammation and leukocyte extravasation. Circ Res.

[132] Schnitzler JG, Poels K, Stiekema LCA, Yeang C, Tsimikas S, Kroon J, Stroes ESG, Lutgens E, Seijkens TTP (2020). Short-term regulation of hematopoiesis by lipoprotein(a) results in the production of pro-inflammatory monocytes. Int J Cardiol.

[133] Shimoni S, Meledin V, Bar I, Fabricant J, Gandelman G, George J (2016). Circulating CD14(+) monocytes in patients with aortic stenosis. J Geriatr Cardiol.

[134] Shirai T, Nazarewicz RR, Wallis BB, Yanes RE, Watanabe R, Hilhorst M, Tian L, Harrison DG, Giacomini JC, Assimes TL, Goronzy JJ, Weyand CM (2016). The glycolytic enzyme PKM2 bridges metabolic and inflammatory dysfunction in coronary artery disease. J Exp Med.

[135] Silvestre-Roig C, Braster Q, Ortega-Gomez A, Soehnlein O (2020). Neutrophils as regulators of cardiovascular inflammation. Nat Rev Cardiol.

[136] Siu SC, Silversides CK (2010). Bicuspid aortic valve disease. J Am Coll Cardiol.

[137] Smith JG, Luk K, Schulz CA, Engert JC, Do R, Hindy G, Rukh G, Dufresne L, Almgren P, Owens DS, Harris TB, Peloso GM, Kerr KF, Wong Q, Smith AV, Budoff MJ, Rotter JI, Cupples LA, Rich S, Kathiresan S, Orho-Melander M, Gudnason V, O'Donnell CJ, Post WS, Thanassoulis G (2014). Association of low-density lipoprotein cholesterol-related genetic variants with aortic valve calcium and incident aortic stenosis. JAMA.

[138] Smith JG, Newton-Cheh C (2015). Genome-wide association studies of late-onset cardiovascular disease. J Mol Cell Cardiol.

[139] Soehnlein O, Libby P (2021). Targeting inflammation in atherosclerosis - from experimental insights to the clinic. Nat Rev Drug Discov.

[140] Song J, Zheng Q, Ma X, Zhang Q, Xu Z, Zou C, Wang Z (2019). Predictive roles of neutrophil-to-lymphocyte ratio and c-reactive protein in patients with calcific aortic valve disease. Int Heart J.

[141] Stansfield BK, Ingram DA (2015). Clinical significance of monocyte heterogeneity. Clin Transl Med.

[142] Steensma DP, Bejar R, Jaiswal S, Lindsley RC, Sekeres MA, Hasserjian RP, Ebert BL (2015). Clonal hematopoiesis of indeterminate potential and its distinction from myelodysplastic syndromes. Blood.

[143] Stephens EH, de Jonge N, McNeill MP, Durst CA, Grande-Allen KJ (2010). Age-related changes in material behavior of porcine mitral and aortic valves and correlation to matrix composition. Tissue Eng Part A.

[144] Stewart BF, Siscovick D, Lind BK, Gardin JM, Gottdiener JS, Smith VE, Kitzman DW, Otto CM (1997). Clinical factors associated with calcific aortic valve disease. Cardiovascular Health Study. J Am Coll Cardiol.

[145] Stewart CR, Stuart LM, Wilkinson K, van Gils JM, Deng J, Halle A, Rayner KJ, Boyer L, Zhong R, Frazier WA, Lacy-Hulbert A, El Khoury J, Golenbock DT, Moore KJ (2010). CD36 ligands promote sterile inflammation through assembly of a Toll-like receptor 4 and 6 heterodimer. Nat Immunol.

[146] Sucosky P, Balachandran K, Elhammali A, Jo H, Yoganathan AP (2009). Altered shear stress stimulates upregulation of endothelial VCAM-1 and ICAM-1 in a BMP-4- and TGF-beta1-dependent pathway. Arterioscler Thromb Vasc Biol.

[147] Syvaranta S, Alanne-Kinnunen M, Oorni K, Oksjoki R, Kupari M, Kovanen PT, Helske-Suihko S (2014). Potential pathological roles for oxidized low-density lipoprotein and scavenger receptors SR-AI, CD36, and LOX-1 in aortic valve stenosis. Atherosclerosis.

[148] Tardif JC, Kouz S, Waters DD, Bertrand OF, Diaz R, Maggioni AP, Pinto FJ, Ibrahim R, Gamra H, Kiwan GS, Berry C, López-Sendón J, Ostadal P, Koenig W, Angoulvant D, Grégoire JC, Lavoie MA, Dubé MP, Rhainds D, Provencher M, Blondeau L, Orfanos A, L'Allier PL, Guertin MC, Roubille F (2019). Efficacy and safety of low-dose colchicine after myocardial infarction. N Engl J Med.

[149] Tawakol A, Migrino RQ, Bashian GG, Bedri S, Vermylen D, Cury RC, Yates D, LaMuraglia GM, Furie K, Houser S, Gewirtz H, Muller JE, Brady TJ, Fischman AJ (2006). In vivo 18F-fluorodeoxyglucose positron emission tomography imaging provides a noninvasive measure of carotid plaque inflammation in patients. J Am Coll Cardiol.

[150] Thanassoulis G, Campbell CY, Owens DS, Smith JG, Smith AV, Peloso GM, Kerr KF, Pechlivanis S, Budoff MJ, Harris TB, Malhotra R, O'Brien KD, Kamstrup PR, Nordestgaard BG, Tybjaerg-Hansen A, Allison MA, Aspelund T, Criqui MH, Heckbert SR, Hwang SJ, Liu Y, Sjogren M, van der Pals J, Kälsch H, Mühleisen TW, Nöthen MM, Cupples LA, Caslake M, Di Angelantonio E, Danesh J, Rotter JI, Sigurdsson S, Wong Q, Erbel R, Kathiresan S, Melander O, Gudnason V, O'Donnell CJ, Post WS (2013). Genetic associations with valvular calcification and aortic stenosis. N Engl J Med.

[151] Thanassoulis G, Massaro JM, Cury R, Manders E, Benjamin EJ, Vasan RS, Cupple LA, Hoffmann U, O'Donnell CJ, Kathiresan S (2010). Associations of long-term and early adult atherosclerosis risk factors with aortic and mitral valve calcium. J Am Coll Cardiol.

[152] Thubrikar MJ, Aouad J, Nolan SP (1986). Patterns of calcific deposits in operatively excised stenotic or purely regurgitant aortic valves and their relation to mechanical stress. Am J Cardiol.

[153] Tousoulis D, Kampoli AM, Tentolouris C, Papageorgiou N, Stefanadis C (2012). The role of nitric oxide on endothelial function. Curr Vasc Pharmacol.

[154] Tsimikas S, Gordts P, Nora C, Yeang C, Witztum JL (2019). Statin therapy increases lipoprotein(a) levels. Eur Heart J.

[155] Tsujioka H, Imanishi T, Ikejima H, Kuroi A, Takarada S, Tanimoto T, Kitabata H, Okochi K, Arita Y, Ishibashi K, Komukai K, Kataiwa H, Nakamura N, Hirata K, Tanaka A, Akasaka T (2009). Impact of heterogeneity of human peripheral blood monocyte subsets on myocardial salvage in patients with primary acute myocardial infarction. J Am Coll Cardiol.

[156] Tzolos E, Andrews JP, Dweck MR (2020). Aortic valve stenosis-multimodality assessment with PET/CT and PET/MRI. Br J Radiol.

[157] U.S. National Library of Medicine (2017) ClinicalTrials.gov. https://clinicaltrials.gov/ct2/show/NCT03051360

[158] U.S. National Library of Medicine (2014) ClinicalTrials.gov. https://clinicaltrials.gov/ct2/show/NCT02109614

[159] U.S. National Library of Medicine (2021) ClinicalTrials.gov. https://clinicaltrials.gov/ct2/show/NCT05162742

[160] U.S. National Library of Medicine (2019) ClinicalTrials.gov. https://clinicaltrials.gov/ct2/show/NCT04023552

[161] van der Heijden C, Groh L, Keating ST, Kaffa C, Noz MP, Kersten S, van Herwaarden AE, Hoischen A, Joosten LAB, Timmers H, Netea MG, Riksen NP (2020). Catecholamines induce trained immunity in monocytes in vitro and in vivo. Circ Res.

[162] van der Heijden T, Kritikou E, Venema W, van Duijn J, van Santbrink PJ, Slütter B, Foks AC, Bot I, Kuiper J (2017). NLRP3 inflammasome inhibition by MCC950 reduces atherosclerotic lesion development in apolipoprotein E-deficient mice-brief report. Arterioscler Thromb Vasc Biol.

[163] van der Valk FM, Bekkering S, Kroon J, Yeang C, Van den Bossche J, van Buul JD, Ravandi A, Nederveen AJ, Verberne HJ, Scipione C, Nieuwdorp M, Joosten LA, Netea MG, Koschinsky ML, Witztum JL, Tsimikas S, Riksen NP, Stroes ES (2016). Oxidized phospholipids on lipoprotein(a) elicit arterial wall inflammation and an inflammatory monocyte response in humans. Circulation.

[164] van der Valk FM, Kuijk C, Verweij SL, Stiekema LCA, Kaiser Y, Zeerleder S, Nahrendorf M, Voermans C, Stroes ESG (2017). Increased haematopoietic activity in patients with atherosclerosis. Eur Heart J.

[165] van Geemen D, Soares AL, Oomen PJ, Driessen-Mol A, Janssen-van den Broek MW, van den Bogaerdt AJ, Bogers AJ, Goumans MJ, Baaijens FP, Bouten CV (2016). Age-dependent changes in geometry, tissue composition and mechanical properties of fetal to adult cryopreserved human heart valves. PLoS ONE.

[166] van Hout GP, Bosch L, Ellenbroek GH, de Haan JJ, van Solinge WW, Cooper MA, Arslan F, de Jager SC, Robertson AA, Pasterkamp G, Hoefer IE (2017). The selective NLRP3-inflammasome inhibitor MCC950 reduces infarct size and preserves cardiac function in a pig model of myocardial infarction. Eur Heart J.

[167] van Kampen E, Jaminon A, van Berkel TJ, Van Eck M (2014). Diet-induced (epigenetic) changes in bone marrow augment atherosclerosis. J Leukoc Biol.

[168] Viney NJ, van Capelleveen JC, Geary RS, Xia S, Tami JA, Yu RZ, Marcovina SM, Hughes SG, Graham MJ, Crooke RM, Crooke ST, Witztum JL, Stroes ES, Tsimikas S (2016). Antisense oligonucleotides targeting apolipoprotein(a) in people with raised lipoprotein(a): two randomised, double-blind, placebo-controlled, dose-ranging trials. Lancet.

[169] Volpato S, Guralnik JM, Ferrucci L, Balfour J, Chaves P, Fried LP, Harris TB (2001). Cardiovascular disease, interleukin-6, and risk of mortality in older women: the women's health and aging study. Circulation.

[170] Xie M, Lu C, Wang J, McLellan MD, Johnson KJ, Wendl MC, McMichael JF, Schmidt HK, Yellapantula V, Miller CA, Ozenberger BA, Welch JS, Link DC, Walter MJ, Mardis ER, Dipersio JF, Chen F, Wilson RK, Ley TJ, Ding L (2014). Age-related mutations associated with clonal hematopoietic expansion and malignancies. Nat Med.

[171] Zeng Q, Song R, Ao L, Xu D, Venardos N, Fullerton DA, Meng X (2014). Augmented osteogenic responses in human aortic valve cells exposed to oxLDL and TLR4 agonist: a mechanistic role of Notch1 and NF-κB interaction. PLoS ONE.

[172] Zeng Q, Song R, Fullerton DA, Ao L, Zhai Y, Li S, Ballak DB, Cleveland JC, Reece TB, McKinsey TA, Xu D, Dinarello CA, Meng X (2017). Interleukin-37 suppresses the osteogenic responses of human aortic valve interstitial cells in vitro and alleviates valve lesions in mice. Proc Natl Acad Sci U S A.

[173] Zhan Q, Zeng Q, Song R, Zhai Y, Xu D, Fullerton DA, Dinarello CA, Meng X (2017). IL-37 suppresses MyD88-mediated inflammatory responses in human aortic valve interstitial cells. Mol Med.

[174] Zhang P, The E, Nedumaran B, Ao L, Jarrett MJ, Xu D, Fullerton DA, Meng X (2020). Monocytes enhance the inflammatory response to TLR2 stimulation in aortic valve interstitial cells through paracrine up-regulation of TLR2 level. Int J Biol Sci.

[175] Zhao TX, Mallat Z (2019). Targeting the immune system in atherosclerosis: JACC state-of-the-art review. J Am Coll Cardiol.

[176] Zheng KH, Tsimikas S, Pawade T, Kroon J, Jenkins WSA, Doris MK, White AC, Timmers N, Hjortnaes J, Rogers MA, Aikawa E, Arsenault BJ, Witztum JL, Newby DE, Koschinsky ML, Fayad ZA, Stroes ESG, Boekholdt SM, Dweck MR (2019). Lipoprotein(a) and oxidized phospholipids promote valve calcification in patients with aortic stenosis. J Am Coll Cardiol.

[177] Zheng KH, Tzolos E, Dweck MR (2020). Pathophysiology of aortic stenosis and future perspectives for medical therapy. Cardiol Clin.

[178] Ziegler-Heitbrock L, Ancuta P, Crowe S, Dalod M, Grau V, Hart DN, Leenen PJ, Liu YJ, MacPherson G, Randolph GJ, Scherberich J, Schmitz J, Shortman K, Sozzani S, Strobl H, Zembala M, Austyn JM, Lutz MB (2010). Nomenclature of monocytes and dendritic cells in blood. Blood.

